# A Master Autoantigen-ome Links Alternative Splicing, Female Predilection, and COVID-19 to Autoimmune Diseases

**DOI:** 10.1101/2021.07.30.454526

**Published:** 2021-08-04

**Authors:** Julia Y. Wang, Michael W. Roehrl, Victor B. Roehrl, Michael H. Roehrl

**Affiliations:** 1 Curandis, New York, USA; 2 Department of Pathology, Memorial Sloan Kettering Cancer Center, New York, USA

## Abstract

Chronic and debilitating autoimmune sequelae pose a grave concern for the post-COVID-19 pandemic era. Based on our discovery that the glycosaminoglycan dermatan sulfate (DS) displays peculiar affinity to apoptotic cells and autoantigens (autoAgs) and that DS-autoAg complexes cooperatively stimulate autoreactive B1 cell responses, we compiled a database of 751 candidate autoAgs from six human cell types. At least 657 of these have been found to be affected by SARS-CoV-2 infection based on currently available multi-omic COVID data, and at least 400 are confirmed targets of autoantibodies in a wide array of autoimmune diseases and cancer. The autoantigen-ome is significantly associated with various processes in viral infections, such as translation, protein processing, and vesicle transport. Interestingly, the coding genes of autoAgs predominantly contain multiple exons with many possible alternative splicing variants, short transcripts, and short UTR lengths. These observations and the finding that numerous autoAgs involved in RNA-splicing showed altered expression in viral infections suggest that viruses exploit alternative splicing to reprogram host cell machinery to ensure viral replication and survival. While each cell type gives rise to a unique pool of autoAgs, 39 common autoAgs associated with cell stress and apoptosis were identified from all six cell types, with several being known markers of systemic autoimmune diseases. In particular, the common autoAg UBA1 that catalyzes the first step in ubiquitination is encoded by an X-chromosome escape gene. Given its essential function in apoptotic cell clearance and that X-inactivation escape tends to increase with aging, UBA1 dysfunction can therefore predispose aging women to autoimmune disorders. In summary, we propose a model of how viral infections lead to extensive molecular alterations and host cell death, autoimmune responses facilitated by autoAg-DS complexes, and ultimately autoimmune diseases. Overall, this master autoantigen-ome provides a molecular guide for investigating the myriad of autoimmune sequalae to COVID-19 and clues to the rare but reported adverse effects of the currently available COVID vaccines.

## Introduction

**Fig. 1.**
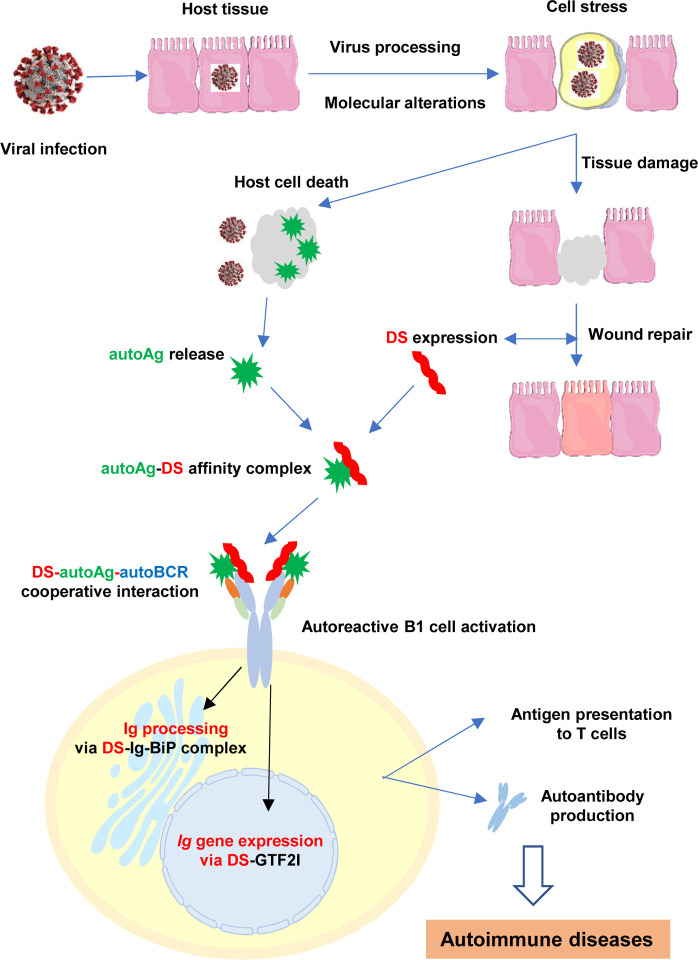
A model on how viral infections lead to autoimmune diseases. Viral infections induce extensive host molecular changes, cell death, and tissue damage. AutoAgs shed from apototic cells form affinity complexes with DS that is overexpressed in the wound area. Cooperative binding of DS-autoAg complexes to autoBCRs activate autoreactive B1 cells. Once internalized via autoBCR, DS engages Ig-processing complexes in the ER and GTF2I in the nucleus to facilitate Ig production. Activated B1 cells secrete autoantibodies and may also present autoAgs to autoreactive T cells, which then leads to autoimmune diseases.

Autoimmune disorders are an important feature of the disease manifestations of COVID-19 and long-COVID syndromes. Based on the insights we gained from numerous COVID-related autoantigens (autoAgs) and their associated cellular process and pathways [1–5], we propose a model to explain how viral infections in general and SARS-CoV-2 in particular can lead to a wide array of autoimmune diseases (Figure 1). We illustrate how viral infections lead to extensive molecular alterations in the host cell, host cell death and tissue injury, autoimmune reactions, and the eventual development of autoimmune diseases.

During infections, opportunistic viruses have to hijack the host cell machinery in order to transcribe and translate the viral genes, synthesize viral proteins with correct polypeptide folding and post-translational modifications, and assemble viral particles. At the same time, viruses have to manipulate the host’s immune defense to avoid elimination. This intricate host-virus symbiosis is accomplished by extensive alterations of host molecules and reprogramming of host molecular networks. The infected host cells undergo extreme stress and ultimately die, which releases altered molecules (i.e., potential autoAgs) that the immune system may recognize as non-self. In response, the host also synthesizes a cascade of molecules such as dermatan sulfate (DS) to facilitate wound healing and dead cell clearance.

We have discovered previously that DS possesses peculiar affinity for apoptotic cells and their released autoAgs [6–9]. DS, a major component of the extracellular matrix and connective tissue, is increasingly expressed during tissue injury and accumulates in wound areas [1, 10]. Because of their affinity, DS and autoAgs form macromolecular complexes which cooperatively activate autoreactive B1 cells. AutoAg-DS complexes may activate B1 cells via a dual binding mode, i.e., with autoAg binding to the variable region of the B1 cell’s autoBCR and DS binding to the heavy chain of the autoBCR. Upon entering B1 cells, DS may regulate immunoglobulin (Ig) production by engaging the Ig-processing complex in the endoplasmic reticulum and the transcription factor GTF2I necessary for Ig gene expression [8, 9]. AutoAg-DS affinity therefore defines a unifying biochemical and immunological property of autoAgs: any self-molecule possessing DS-affinity has a high propensity to become autoantigenic, and this has led to the identification of numerous autoAgs [7, 11–13].

To gain a better understanding of autoimmune sequelae due to COVID-19, we present a master autoantigen atlas of over 750 potential autoAgs identified from six human cell types [1, 2, 4, 5, 7, 11]. These autoAgs show significant correlation with pathways and processes that are crucial in viral infection and mRNA vaccine action, reveal common autoAgs associated with apoptosis and cell stress which may serve as markers for systemic autoimmune diseases, and provide a detailed molecular map for understanding and for investigating diverse autoimmune sequalae of COVID-19 and potential rare side-effects to viral vector- and mRNA-based vaccines. For the first time, we reveal intriguing features of autoAgs and their coding genes. Furthermore, we discuss how UBA1 (or UBE1, ubiquitin-like modifier-activating enzyme 1), an autoAg found overexpressed in SARS-CoV-2 infection, may predispose aging females to autoimmune disorders.

## Results and Discussion

### The master autoantigen-ome

To understand the diversity of autoimmune diseases, we were curious to know how many autoAgs possibly exist. A total of 751 potential autoAgs were identified (Table 1) when we combined all DS-affinity autoAgs profiled from six human cell lines, namely, HFL1 fetal lung fibroblasts, HEp2 fibroblasts, A549 lung epithelial cells, HS-Sultan and Wil2-NS B-lymphoblasts, and Jurkat T-lymphoblasts. Extensive literature searches confirmed that at least 400 of these proteins (53.3%) have been reported as targets of autoantibodies in a wide variety of autoimmune diseases and cancer (see autoAg confirmation references in Table 1). The majority of unconfirmed or putative autoAgs are isoforms of or structurally similar to reported autoAgs and are yet-to-confirmed autoAgs. For example, 56 ribosomal proteins were identified by DS-affinity, but only 22 are thus far confirmed autoAgs; but given their structural similarity and shared epitopes, it is likely that most if not all of the 56 ribosomal proteins are likely true autoAgs awaiting further confirmation.

The master autoantigen-ome contains clusters of protein families, including 56 ribosomal proteins, 27 proteasome subunits, 19 heterogeneous ribonucleoproteins, 17 splicing factors, 17 ATP-dependent RNA helicase subunits, 16 eukaryotic translation initiation factors, 16 histones, 16 aminoacyl-tRNA synthases, 12 heat shock proteins, 9 elongation factors, 9 small nuclear ribonucleoproteins, 8 T-complex protein 1 subunits, and 7 14-3-3 proteins. In addition, there are multiple isoforms of numerous proteins, such as actin, tropomyosin, myosin, collagen, tubulin, and annexin.

**Fig. 2.**
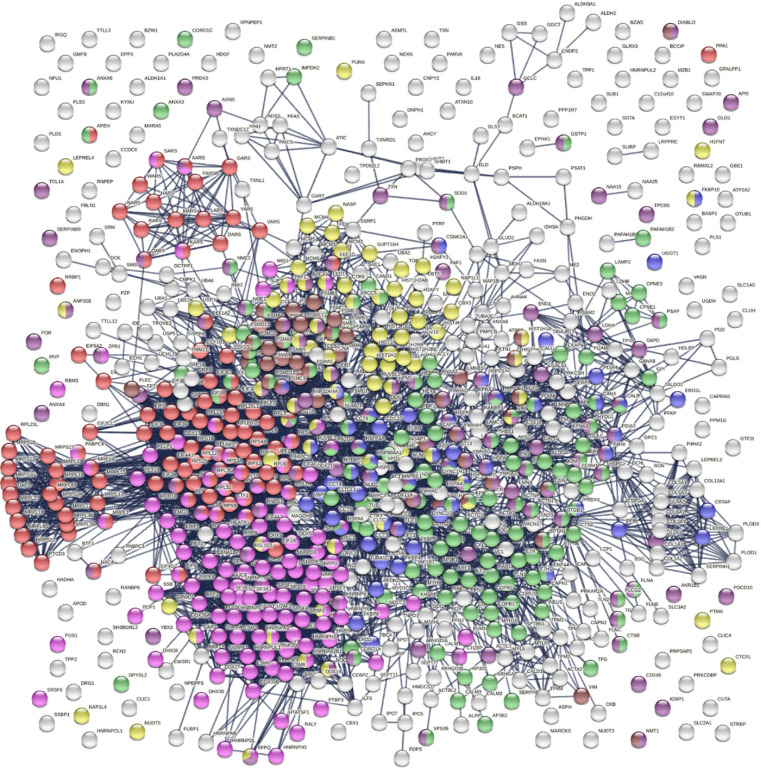
The master autoAg-ome of 751 DS-affinity proteins identified from 6 cell types forms a highly interacting connected network. Lines represent protein-protein interactions with the highest confidence cutoff. Colored proteins are associated with translation (104 proteins, red), RNA processing (120 proteins, pink), protein folding (53 proteins, blue), vesicle-mediated transport (141 proteins, green), chromosome organization (76 proteins, yellow), regulation of cell death (110 proteins, dark purple), and apoptosis (46 proteins, brown).

**Fig. 3.**
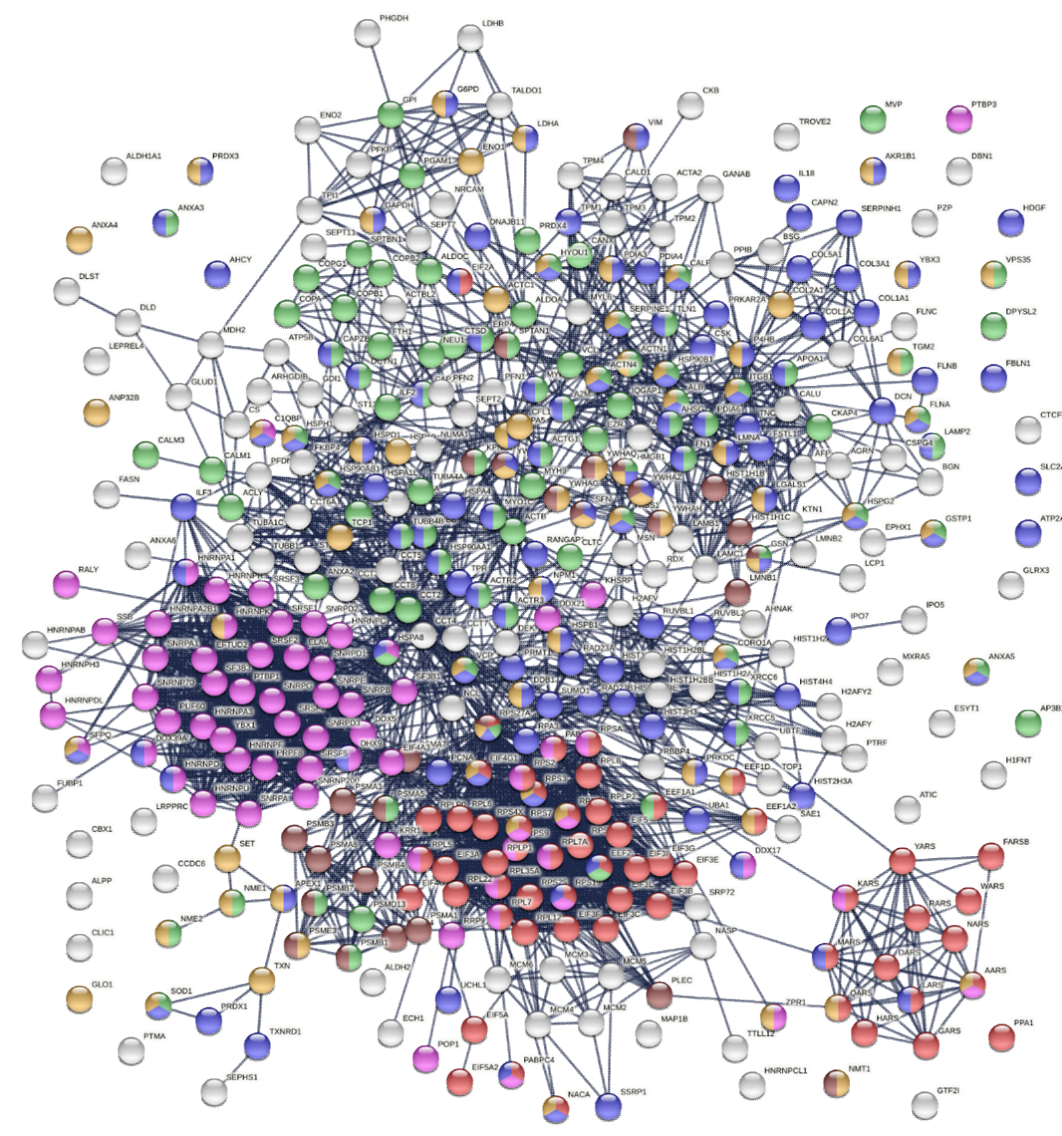
Protein interaction network of the 400 confirmed autoAgs. Lines represent protein-protein interactions with highest confidence. Colored proteins are associated with translation (57 proteins, red), RNA processing (65 proteins, pink), vesicle-mediated transport (89 proteins, green), response to stress (125 proteins, blue), regulation of cell death (74 proteins, amber), and apoptosis (28 proteins, brown).

The 751 confirmed and putative autoAgs are highly connected and have significantly more interactions than what would be expected for a random set of proteins of similar size drawn from the genome (exhibiting 6,936 interactions vs. 3,596 expected with the highest confidence level cutoff; enrichment p value <1.0e-16) as per protein-protein interaction analysis in STRING [14] (Fig. 2). The 400 confirmed autoAgs also form a similar, strong interacting network (exhibiting 2,758 interactions vs. 1,269 expected; enrichment p value <10e-16) (Fig. 3). The tight connections within the autoAg network suggest that these proteins are biologically connected, and given that they are all identified by DS-affinity, the autoAg protein networks offer a glimpse of the biological roles and functions of DS that await further investigation.

**Fig. 4.**
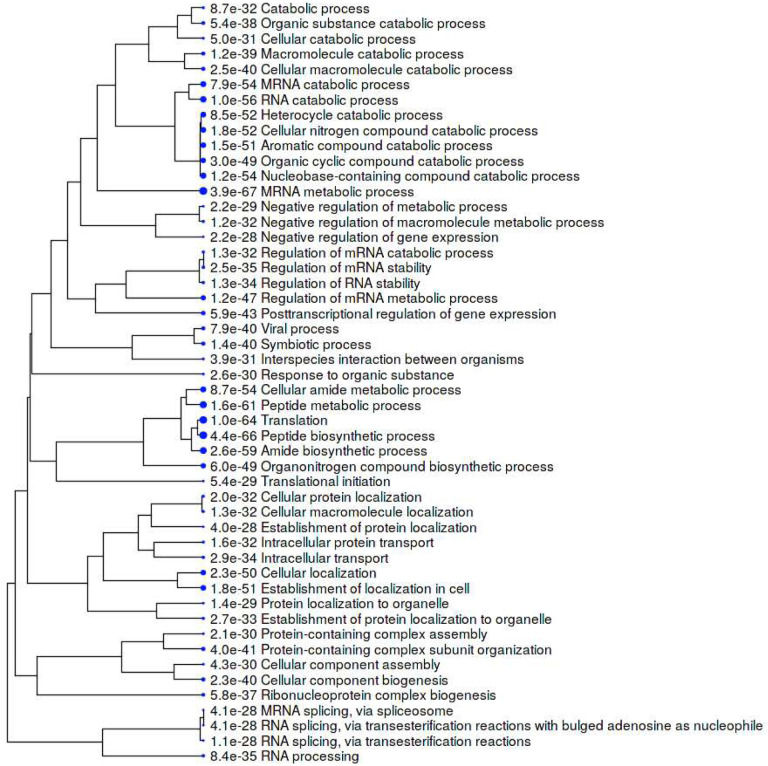
Hierarchical clustering of the top 50 GO Biological Processes associated with the master autoantigen-ome of 751 DS-affinity autoAgs. Bigger blue dots indicate more significant p values.

The 751-protein master autoantigen-ome is significantly associated with many biological processes and pathways, most notably translation, RNA processing, RNA splicing, protein folding, vesicle-mediated transport, chromosome organization, regulation of cell death, and apoptosis (Figs. 2 and 4). The 400 confirmed autoAgs are similarly significantly associated with the same processes and pathways (Fig. 3). In addition, these proteins are associated with numerous other processes, e.g., mRNA metabolic process, peptide metabolic process, establishment of localization in the cell, intracellular transport, interspecies interaction between organisms, viral process (infection and virulence), symbiotic process, and response to stress (Figs. 2–4). Hierarchical clustering [15] of the top 50 enriched Gene Ontology Biological Processes reveals RNA processing, particularly RNA splicing, to be the most noticeable (Fig. 4).

### The COVID-19 autoantigen-ome

To find out how many autoAgs in the autoantigen-ome are potentially affected by SARS-CoV-2 infection, we looked for them in currently available multi-omic COVID data compiled by Coronascape [16–37]. Remarkably, 657 (87.5%) proteins of the 751-member master autoantigen-ome are found to be affected in SARS-CoV-2 infection (Table 1 and Supplemental Table 1). Among them, 109 proteins were found up-regulated only, 176 were found down-regulated only, and 343 were found both up- and down-regulated at protein and/or RNA levels in virally infected cells or COVID-19 patients (Table 1 and Fig. 6). In addition, 191 potential autoAgs were found in the interactomes of different SARS-CoV-2 viral component proteins, meaning that they may directly or indirectly interact with the virus.

**Fig. 5.**
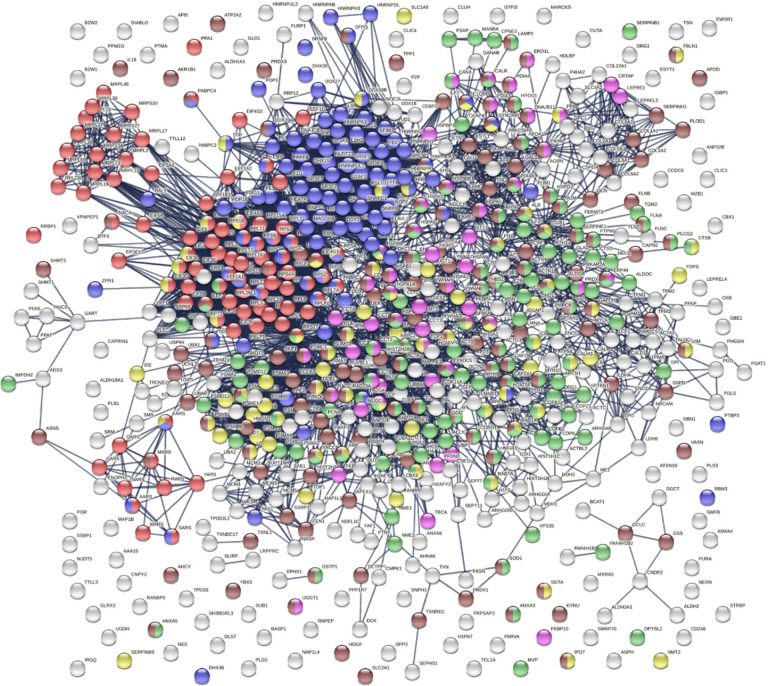
The COVID autoantigen-ome of 657 autoAg candidates. Lines represent protein-protein interactions with highest confident level. Colored proteins are associated with translation (87 proteins, red), RNA processing (103 proteins, blue), protein folding (51 proteins, pink), symbiont process (78 proteins, yellow), vesicle-mediated transport (125 proteins, green), and response to stress (161 proteins, brown).

**Fig. 6.**
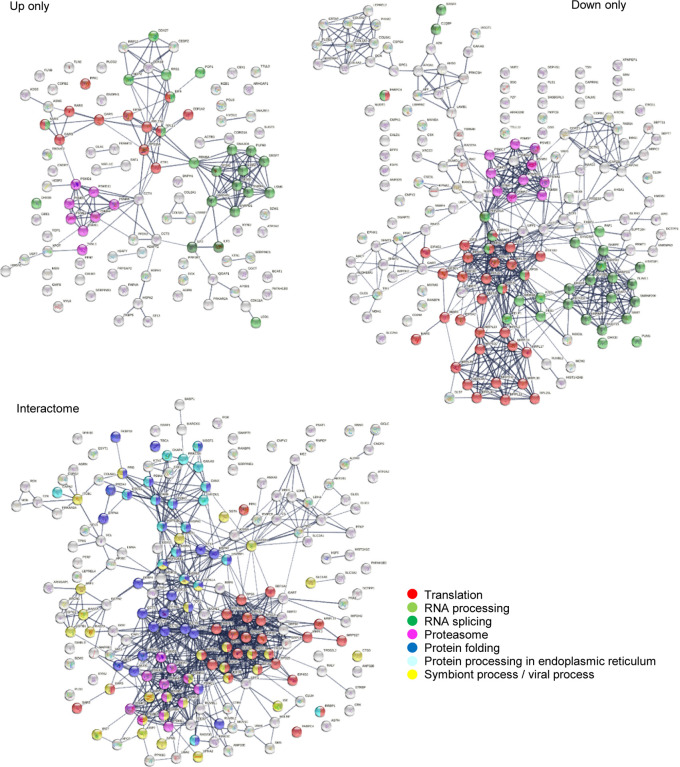
COVID-affected autoAgs that are found up-regulated only, down-regulated only, or interacting with SARS-Cov-2 proteins. Note the significant enrichment of proteins associated with translation, RNA processing and splicing, and other processes.

The 657-member COVID autoantigen-ome is also a highly interacting protein network (Fig. 5). Not surprisingly, these proteins are significantly associated with processes that are crucial in viral infection, e.g., RNA processing, mRNA metabolic process, regulation of mRNA stability, translation, peptide biosynthetic process, protein folding, intracellular transport, vesicle-mediated transport, regulated exocytosis, symbiont process, and interspecies interaction between organisms, response to stress, regulation of cell death, and apoptosis (Fig. 5). We also analyzed the 109 up-only and the 176 down-only protein networks separately. Both networks are significantly associated with translation, RNA processing and splicing, and the proteasome, which further illustrates that these processes are perturbed by the viral infection (Fig. 6).

Translation is an essential step in viral replication and mRNA vaccine action. DS-affinity identified 19 eukaryotic translation initiation factors, with 15 thus far being confirmed autoAgs (Table 1). In particular, 8 of the 13 subunits of the human eIF3 complex were found in the interactome of the NSP1 protein of SARS-CoV-2, and all 8 are known autoAgs (Table 1). eIF3 is essential for the most forms of cap-dependent and cap-independent translation initiation and stimulates nearly all steps of translation initiation, as well as other phases of translation such as recycling. eIF3 functions in a number of prominent human pathogens, e.g., HIV and HCV; and the present finding indicates that eIF3 also functions in SARS-CoV-2 infection.

Among the 657 COVID-affected DS-affinity proteins, 369 (56%) are thus far confirmed autoAgs, accounting for 92% of the 400 confirmed autoAgs of the master autoantigen-ome. This vast number of perturbed autoAgs demonstrates that COVID-19 could lead to a wide variety of autoimmune diseases. For example, 42 autoAgs are associated with the myelin sheath and many are associated with other components of the nervous system, as we have described previously, which may help explain a myriad of neurological symptoms caused by COVID-19 [1]. As another example, 11 autoAgs are related to stress fibers (contractile actin filament bundles consisting of short actin filaments with alternating polarity) and 25 proteins are associated with myofibrils (contractile elements of skeletal and cardiac muscle), which may explain various muscular and cardiomuscular sequelae of COVID-19.

A few autoAgs also interact with multiple viral proteins of SARS-CoV-2, suggesting that they play important roles in COVID-19 and merit further investigation. For example, ESYT1 and MOV10 interact with 12 viral proteins, CALU interacts with 11, HSPA5 interacts with 9, COPG1 and ARHGAP1 interact with 8, PLD3 and MARCKS interact with 7, and IDE interacts with 6 viral proteins (Table 1). PLD3 (a phospholipase) influences the processing of amyloid-beta precursor protein and is associated with spinocerebellar ataxia and Alzheimer’s disease. IDE (insulin-degrading enzyme) degrades intracellular insulin and is associated with diabetes.

### AutoAg coding gene characteristics and alternative splicing

**Fig. 7.**
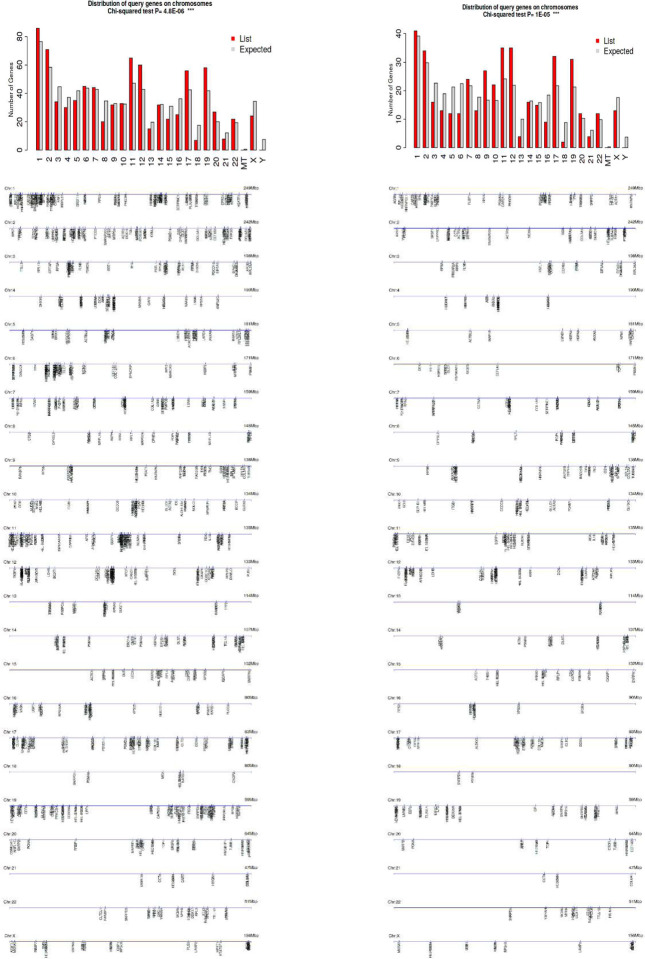
Distribution of autoAg coding genes by chromosomes. Left: 751 confirmed and putative autoAgs. Right: 400 confirmed autoAgs only.

To further understand the autoantigen-ome, we mapped the coding genes for 751 proteins of the master autoantigen-ome, and they are distributed over all chromosomes (Fig. 7). Since these include both confirmed and putative autoAgs, one may argue that some of the putative autoAgs may not be true and the gene characteristics may not be meaningful. Therefore, we also mapped the genes for the 400 confirmed autoAgs, and they are similarly distributed over all chromosomes (Fig. 7). For both confirmed and putative autoAgs, coding gene prevalence is significantly higher on chromosomes 11, 12, 17, and 19, lower on chromosome 18, and almost absent on chromosome Y (Fig. 7). Various cluster loci are noticeable, e.g., on chromosomes 1, 11, 12, 17, and 19.

**Fig. 8.**
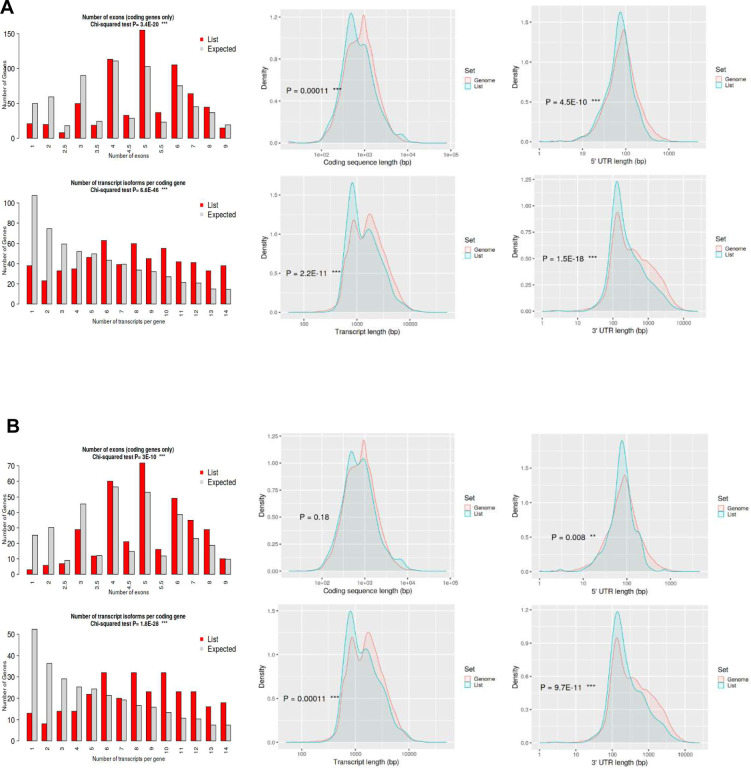
Characteristics of the autoAg coding genes compared with the rest in the genome. Differences are evaluated with Chi-squred and Student’s t-tests. (**A**) 751 confirmed and putative autoAgs. (**B**) 400 confirmed autoAgs.

Intriguingly, autoAg coding genes contain significantly larger numbers of exons than expected, with the majority containing at least 4 exons (Fig. 8). The number of transcript isoforms per coding gene is also significantly skewed towards higher numbers, and those with ≥6 isoforms are particularly dominant. Furthermore, the lengths of coding sequence, transcript, and 3’ and 5’-UTR of autoAg coding genes are skewed towards shorter sizes relative to the distribution of all coding genes (Fig. 8). We also examined the coding genes of the 400 confirmed autoAgs, and they show similar dominance in higher number ofexons and -isoforms, shorter transcripts, and shorter 3’-UTR lengths (Fig. 8).

The predominance of multiple exons and transcript variants suggests a role for RNA processing and alternative splicing in the origination of autoAgs. For genes with multiple exons, alternative splicing can yield a range of unique protein isoforms by varying the exon composition. Curiously, numerous components of the splicing machinery are well-known nuclear autoAgs. In fact, this study identified 120 potential autoAgs associated with RNA processing and 70 potential autoAgs associated with RNA splicing (Table 1 and Figs. 2–3). The majority of these have been found to be affected by SARS-CoV-2 infection (Figs. 5–6).

During splicing, a group of snRNPs (small nuclear ribonucleoproteins) bind to the intron of a newly formed pre-mRNA and splice it to result in a mature mRNA. Ten snRNP autoAgs are identified by DS-affinity, 8 of which have been found to be affected by SARS-CoV-2 infection (Table 1). During splicing, snRNAs undergo conformational rearrangements that are catalyzed by the DEAH/DEAD box superfamily of RNA helicases. 11 such helicases are identified by DS-affinity, and 10 have been found to be affected by the viral infection (Table 1). Serine/arginine-rich splicing factors, such as SRSF1 (also known as alternative splicing factor 1), are sequence-specific splicing factors involved in pre-mRNA splicing. 9 SRSF proteins are identified by DS-affinity, with 7 found to be affected by the viral infection. Seven additional splicing factors are identified by DS-affinity (e.g., poly(U)-binding splicing factor PUF60), with all found to be affected by SARS-CoV-2 infection. Heterogeneous nuclear ribonucleoproteins (hnRNPs) play various roles in gene transcription and post-transcriptional modification of pre-mRNA, e.g., binding pre-mRNAs to render splice sites more or less accessible to the spliceosome and suppressing RNA splicing at a particular exon. 19 hnRNP proteins are identified by DS-affinity, with 17 found affected by SARS-CoV-2 infection.

The large number of autoAgs of the RNA splicing machinery and their involvement in SARS-CoV-2 infection provide support to the notion that viral infections exploit alternative splicing. It is logical to speculate that viruses hijack the splicing machinery to force the host to synthesize virus-beneficial protein isoforms and thereby reprogram the host cellular protein network so that the virus can survive and replicate. It is also plausible that protein isoforms from virus-induced alternative splicing are recognizable by our immune system as unusual and non-self and hence may trigger an (auto)immune response.

Various studies have reported alternative splicing among autoAgs. For example, an informatics analysis of 45 autoAgs showed that alternative splicing occurred in 100% of the transcripts, which was significantly higher than the ~42% rate observed in a randomly selected set of 9,554 gene transcripts. Furthermore, 80% of the transcripts underwent non-canonical alternative splicing, which was significantly higher than the <1% rate in randomly selected human gene transcripts [38]. As another example, Ro52/SSA is one of the autoAg targets strongly associated with the autoimmune responses in mothers whose children have manifestations of neonatal lupus. The gene for full-length Ro52 spans 10 kb of DNA and contains 7 exons, and an alternatively spliced transcript encoding a novel autoAg expressed in the fetal and adult heart has been identified [39]. In a patient with primary Sjörgren syndrome, an alternative mRNA variant of the nuclear autoAg La/SSB was found to result from a promoter switch and alternative splicing [40].

### Common autoAgs associated with cell stress and apoptosis

**Fig. 9.**
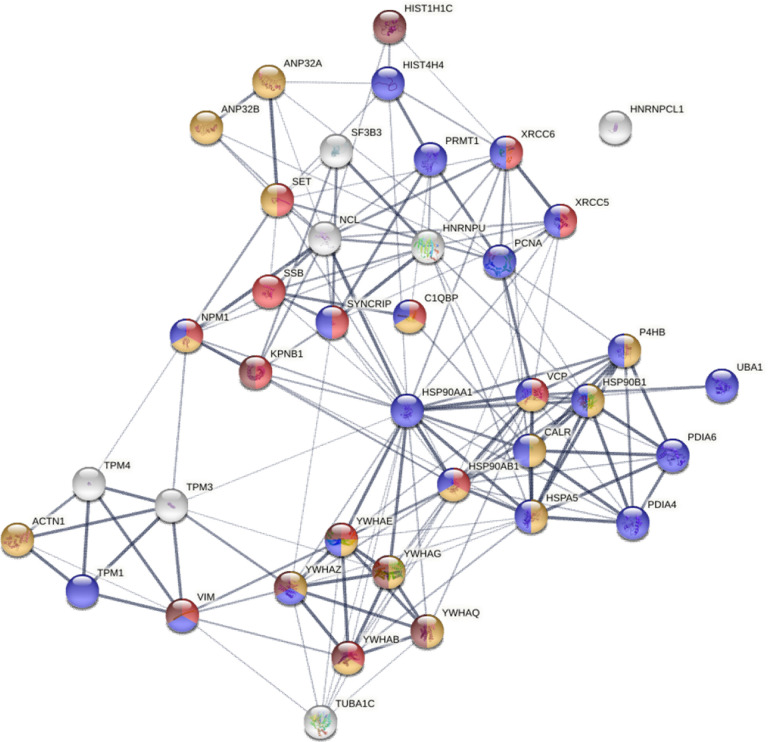
Common autoAgs identified from all six cell types examined in this study. Colored are proteins associated with viral infection (13 proteins, red), regulation of apoptotic process (17 proteins, amber), response to stress (22 proteins, blue), and apoptosis (8 proteins, brown).

We have consistently found that DS binds apoptotic cells regardless of cell type [6, 8]. To figure out which molecules are involved in this affinity, we searched for DS-affinity proteins shared in all 6 human cell lines of this study and found 39 autoAg candidates (Fig. 9). These include 9 ER chaperone complex proteins, 5 14-3-3 proteins, 3 hnRNPs, and 3 tropomyosin proteins. All are known autoAgs except for ANP32A and YWHAB (14-3-3 alpha/beta). Given that ANP32A’s paralog ANP32B and 5 other 14-3-3 isoforms are known autoAgs, it is likely they are also true autoAgs. Remarkably, several classical ANA (antinuclear antibody) autoAgs that define systemic autoimmune diseases are among the autoAgs found in the DS-affinity proteomes of all 6 human cell lines, including histone H1 and H4, SSB (lupus La), XRCC5/Ku80, XRCC6/Ku70, and PCNA. Because these autoAgs are commonly found in apoptotic cells, it is not surprising that autoimmune responses targeting these autoAgs tend to be systemic; in other words, they all are potential markers of systemic autoimmune diseases.

Based on GO Biological Process and Reactome Pathway analysis, 22 of the common autoAgs are associated with cellular responses to stress, 17 are associated with regulation of apoptotic processes, and 8 are markers of apoptosis (Fig. 9). Moreover, these common autoAgs are involved in chromosome organization (ANP32A, ANP32B, H1–2, H4, KPNB1, NPM1, PCNA, SET, XRCC5, XRCC6), cytoskeleton organization (ACTN1, CALR, TPM1, TPM3, TPM4, TUBA1C, VIM), and mitochondrial membrane organization (YWHAB, YWHAE, YWHAG, YWHAQ, YWHAZ). These findings reveal that apoptosis is accompanied by reorganization of the nucleus, mitochondria, and cytoskeleton.

Furthermore, 37 of the 39 common autoAgs were altered in SARS-CoV-2 infection. Based on GO Biological Process analysis, 13 of these proteins are involved in viral processing, namely, KPNB1, C1QBP, HSP90AB1, NPM1, SYNCRIP, SET, SSB, XRCC5, XRCC6, VCP, VIM, YWHAB, and YWHAE. These findings further support our model of linking viral infection to autoimmunity, with viral infections leading to host cell stress, cell death, autoimmune reactions, and eventually autoimmune diseases (Fig. 1).

### UBA1, X-inactivation escape, and female predilection of autoimmunity

Among the above common autoAgs, UBA1 (or UBE1, ubiquitin-like modifier-activating enzyme 1) plays an essential role in dead cell clearance. UBA1 catalyzes the first step in ubiquitination – the “kiss of death” – that marks cellular proteins for degradation. It has long been speculated that dysregulation of apoptotic pathways and dysfunctional clearance of dead cells are among the main causes of autoimmunity, which is in line with our findings [6, 8]. Apoptosis also directly contributes to the maintenance of lymphocyte homeostasis and the deletion of autoreactive cells. Therefore, dysfunction of UBA1 could result in deficient clearance of apoptotic cells and aberrant autoimmunity.

Recently, UBA1 somatic mutations have been linked to a severe adult-onset autoinflammatory disease termed VEXAS syndrome [41]. A somatic mutation affecting methionine-41 in UBA1 results in a loss of the canonical cytoplasmic isoform of UBA1 and in the expression of a novel catalytically impaired isoform. Additionally, mutant peripheral blood cells show decreased ubiquitination and activated innate immune pathways.

**Fig. 10.**
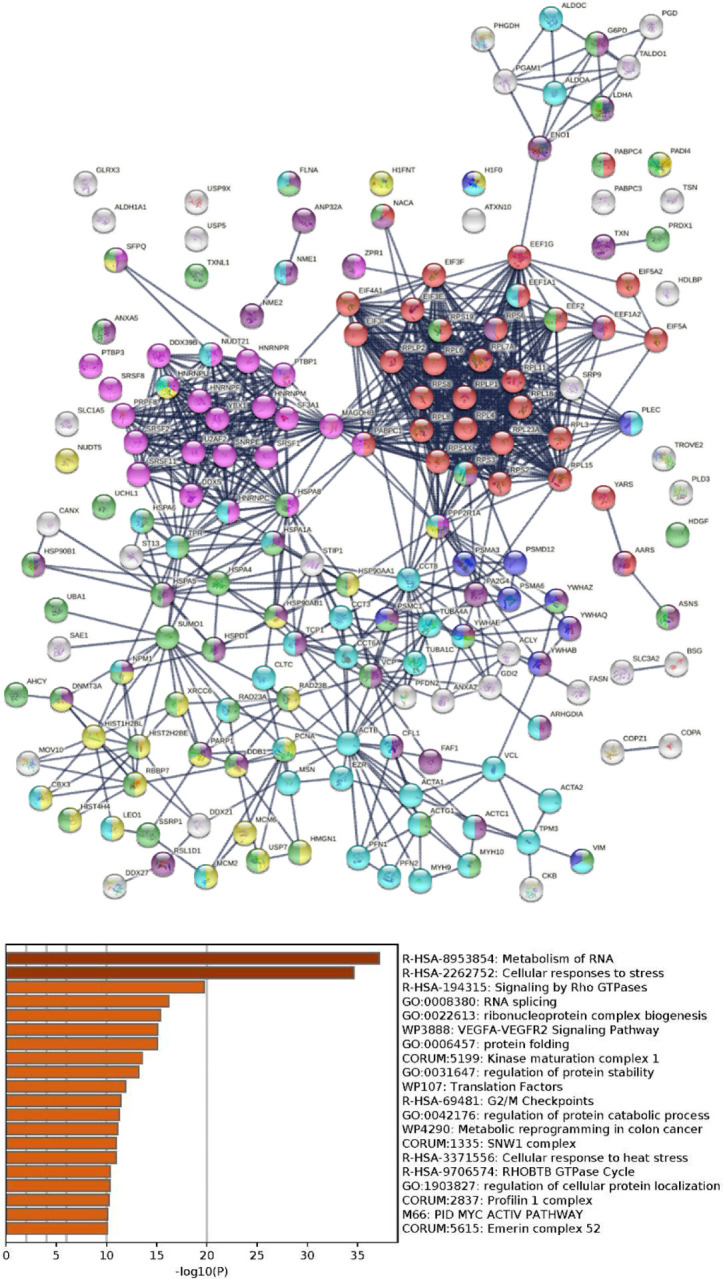
**Top:** Potential autoAgs affected by ubiquitination in SARS-Cov-2 infection (lines represent protein-protein interactions with the highest confidence). Colored are proteins associated with translation (32 proteins, red), RNA splicing (25 proteins, pink), regulation of cell death (40 proteins, dark purple), chromosome organization (26 proteins, yellow), response to stress (50 proteins, green), cytoskeleton (45 proteins, aqua), and apoptosis (11 proteins, blue). **Bottom:** Top 20 enriched processes and pathways associated with the ubiquitinated autoAgs.

Strikingly, UBA1 protein expression is found up-regulated at different time points of SARS-CoV-2 infection, whereas two deubiquitinating enzymes, USP9X and USP5, are down-regulated [33] (Supplemental Table 1). Furthermore, among the 657 proteins of the COVID autoantigen-ome, 178 have been found to be affected by ubiquitination (Fig. 10). They are most significantly associated with RNA metabolism and cellular response to stress. In addition, ubiquitination affects proteins involved in signaling by Rho GTPase, RNA splicing, translation, protein folding, nonsense-mediated decay, DNA damage stress-induced senescence, and the cytoskeleton. These findings underline the extensive involvement of ubiquitination in viral infection.

UBA1 is coded by the *UBA1* gene located on the X chromosome with no homolog on the Y chromosome, and more importantly, *UBA1* can escape X-chromosome inactivation. *UBA1* appears to be protected against chromosome-wide transcriptional silencing by a chromatin boundary flanked by histone H3 modifications and CpG hypomethylation [42]. In human female fibroblasts, UBA1 mRNA is detected from both the active and inactive X chromosomes, and UBA1 is expressed in a large panel of somatic cell hybrids retaining inactive X chromosomes [43]. In human endothelial cells from dizygotic twins, UBA1 and a few other X-chromosome encoded proteins are expressed at higher levels in female cells [44]. UBA1 expression is estimated to be ~ 60% from X-active alleles, 30% biallelic, and 10% from X-inactive alleles [45].

X-linked genes, particularly escape genes, contribute to sex differences. In women, about 15% of X-linked genes are bi-allelically expressed, and expression from the inactive X allele varies from a few percent to near equal to that of the active allele [46]. X-inactivation and escape may enhance phenotypic differences between females and males and may also enhance variability within females due to mosaicism from cells with the X-maternal or X-paternal inactivated and to a variable degree of escape from X-inactivation [46]. Aging, which is associated with telomere shortening, can relax X-inactivation and force global transcriptome alterations [47], which may lead to gene escape and altered expression of UBA1. Therefore, dysfunction of UBA1 due to X-inactivation escape may predispose women, particularly aging women, to increasing dysfunctional regulation of apoptosis and aberrant autoimmunity.

### Considerations for vaccine design based on Spike-protein via viral vectors or mRNAs

To understand the various rare but reported side effects from the currently available viral vector- and mRNA-encoded S-protein COVID vaccines, we searched for autoAgs that may interact with the spike protein of SARS-CoV-2 and found 15 autoAg candidates (Table 2). Of these, CALU, ESYT1, MOV10, and MARCKS may also interact with many other SARS-CoV-2 proteins as discussed earlier. Curiously, at least 2 of these are associated with blood clotting problems, and 5 are implicated in neurological disorders (Table 2). For example, CALU (calumenin) is a calcium-binding protein and is expressed in high levels in the heart, placenta, and skeletal muscle. CALU is associated with pharmacodynamics and response to elevated platelet cytosolic Ca^2+^, platelet degranulation, and Coumarin/Warfarin resistance. Warfarin is an anticoagulant (blood thinner) drug used to treat blood clots such as deep vein thrombosis and pulmonary embolism and to prevent stroke in people with heart problems such as atrial fibrillation, valvular heart disease or in people with artificial heart valves.

Although largely speculative at present, these potential S-protein-interacting autoAgs may provide partial explanations for the rare hematological, neurological, and muscular side effects reported for the currently available COVID vaccines (Table 2). Although it is known that S proteins are synthesized intracellularly following vaccination with mRNAs or viral vectors, many of the precise molecular steps remain unknown. In particular, how do these newly synthesized S proteins fold and are they glycosylated differently depending on the cell type that rakes up the mRNA or the viral vector? How does the newly synthesized S protein interact with other host cell components before being processed (or degraded) and presented to immune cells? For example, could the nascent S proteins interact with CALU or ESYT1 to cause blood clotting problems, could S protein interaction with HSPA5 contributes to fungal infection outbreaks as seen in India? These and many other questions await further investigation. This is of interest because mRNA and vector-based vaccines make use of a variety of cell types in vivo to produce the immunogen, whereas recombinant protein-based vaccines introduce the ex vivo prepared immunogen directly to the immune system.

In addition, this study identified a large number of autoAg candidates that are crucial for vector-based or mRNA vaccine action, including translation, RNA processing and metabolism, vesicles and vesicle-mediated transport, and protein processing and transport (Figs. 2–6). For example, the master autoantigen-ome contains 56 ribosomal proteins, 16 eukaryotic translation initiation factors, 16 aminoacyl-tRNA synthases/ligases, and 6 translation elongation factors, all of which are essential actors in translating mRNAs into proteins. There are also many autoAgs related to protein folding and post-translational protein modification, although it is not clear whether the S proteins are folded and post-translationally modified before being processed and presented to immune cells in the currently used mRNA or vector vaccines for COVID-19. These potential autoAgs may confer clues to understanding the observed rare adverse events and should help guide the future development of even safer vaccines.

### Conclusion

In this report, we compiled a master autoantigen-ome of 751 potential autoAgs, 657 of which are affected in SARS-CoV-2 infection, and 400 of which are confirmed autoAgs in a wide variety of autoimmune diseases and cancer. Our proposed model (Fig. 1) provides a plausible explanation for how a cascade of molecular changes associated with viral infection leads to cell stress, apoptosis, and subsequent autoimmune responses. The large number of autoAg candidates associated with SARS-CoV-2 infection provides a mechanistic rationale for the close monitoring of autoimmune diseases that may follow the COVID-19 pandemic. In addition, the coding gene characteristics of autoAgs described in this study provide further insights into the genetic origination of autoAgs. The significance of ubiquitination in apoptotic cell clearance and protein turnover and the X-linked escape expression of UBA1 might explain, in part, the predisposition of aging women to autoimmune diseases.

## Materials and Methods

### DS-affinity autoAg identification

Potential autoAgs were identified by DS-affinity from protein extracts from six human cell lines as previously described, including HFL1 fetal lung fibroblasts [1], A549 lung epithelial cells [2], HS-Sultan B-lymphoblasts [4], Wil2-NS B-lymphoblasts [7], Jurkat T-lymphoblasts [5], and HEp-2 fibroblasts [11].

### Autoantigen literature text mining

Each DS-affinity protein was verified as to whether it is a target of autoantibodies by an extensive literature search on PubMed. Search keywords included the MeSH keyword “autoantibodies”, the protein name or its gene symbol, or alternative names and symbols. Only proteins for which specific autoantibodies are reported in PubMed-listed journal articles were considered “confirmed” or “known” autoAgs in this study.

### COVID data comparison

DS-affinity proteins were compared with currently available COVID-19 multi-omic data compiled in the Coronascape database (as of 05/27/2021) [16–37]. These data have been obtained with proteomics, phosphoproteomics, interactome and ubiquitome studies, and RNA-seq techniques. Up- and/or down-regulated proteins or genes were identified by comparing cells infected vs. uninfected by SARS-CoV-2 or COVID-19 patients vs. healthy controls. Similarity searches were conducted to identify DS-affinity proteins that are similar to those found up- and/or down-regulated in the viral infection at any omic level.

### Protein network analysis

Protein-protein interactions were analyzed with STRING [14]. Interactions include both direct physical interaction and indirect functional associations, which are derived from genomic context predictions, high-throughput lab experiments, co-expression, automated text mining, and previous knowledge in databases. Each interaction is annotated with a confidence score between 0 (lowest) and 1 (highest), indicating the likelihood of an interaction to be true. Enrichment of pathways and processes were analyzed with Metascape [16], which utilize various ontological sources such as KEGG Pathway, GO Biological Process, Reactome Gene Sets, and Canonical Pathways. All genes in the genome were used as the enrichment background. Terms with a p value <0.01, a minimum count of 3, and an enrichment factor (ratio between the observed counts and the counts expected by chance) >1.5 were grouped into clusters based on their membership similarities. The most statistically significant term within a cluster was chosen to represent the cluster.

### Gene characteristic analysis

Gene characteristics were analyzed with ShinyGO [15]. ShinyGO is based on a large annotation database derived from Ensembl and STRING-db. The characteristics of the genes for the groups of autoAgs in this study were compared with the rest in the genome. Chi-squared and Student’s t-tests were run to see if the autoAg genes had special characteristics when compared with all other genes in the human genome.

## Supplementary Material

Supplement 1

## Figures and Tables

**Table 1.** Autoantigens identified by DS-affinity and their alterations in SARS-CoV-2 infection

P	Gene	Protein	Cell line						SARS-Cov-2 infection			DS affinity		Ref.
			HFL1	HS-Sultan	Wil2	A549	Jurkat	HEp-2	u	d	interact.	hi	low	
5	A2M	Alpha-2-macroglobulin	+			+				d			+	[1]
6	AARS	Alanine-tRNA ligase, AARS1	+	+					u	d			+	[2]
15	ACLY	ATP-citrate synthase		+		+			u	d			+	[3]
4	ACTA1	Actin, alpha skeletal muscle			+				u	d		+		[4]
10	ACTA2	Actin, aortic smooth muscle	+	+		+		+	u	d		+	+	[5]
8	ACTB	Actin, cytoplasmic 1	+	+		+		+	u	d		+	+	[6]
7	ACTBL2	Beta-actin-like protein	+	+		+		+	u	d		+	+	[6]
2	ACTBL3	Putative beta-actin-like protein 3, kappa actin, POTEKP			+		+		u				+	
6	ACTC1	Actin, alpha cardiac muscle					+		u	d		+	+	[7]
4	ACTG1	Actin, cytoplasmic 2			+		+		u	d		+	+	[8]
28	ACTN1	Alpha-actinin-1	+	+	+	+	+	+	u	d			+	[9]
22	ACTN4	Alpha-actinin-4	+	+	+	+		+	u	d			+	[5]
2	ACTR2	Actin-related protein 2		+					u	d			+	[10]
2	ACTR3	Actin-related protein 3		+					u				+	[11]
2	ADSS2	Adenylosuccinate synthetase isozyme 2, ADSS				+			u				+	
3	AFP	Alpha-fetoprotein	+	+		+				d			+	[12]
2	AGRN	Agrin				+			u		Nsp6 Nsp13 Orf8 Orf10	+		[13]
15	AHCY	Adenosylhomocysteinase, SAHH		+		+				d			+	[14]
5	AHNAK	Neuroblast differentiation-associated protein	+						u	d			+	[15]
4	AHSA1	Activator of 90 kDa heat shock protein ATPase homolog 1		+						d			+	
2	AHSG	Alpha-2-HS-glycoprotein, FETUA						+		d			+	[16]
5	AKR1B1	Aldo-keto reductase family 1 member B1				+			u	d	Orf3		+	[17]
10	ALB	Albumin	+			+			u	d		+	+	[18]
5	ALDH18A1	Delta-1-pyrroline-5-carboxylate synthetase		+			+			d			+	
23	ALDH1A1	Retinal dehydrogenase 1				+			u	d			+	[19]
5	ALDH2	Aldehyde dehydrogenase, mitochondrial				+			u	d	E Nsp5 Nsp12 Nsp16		+	[20]
5	ALDH3A1	Aldehyde dehydrogenase 3, ALDH3				+			u	d			+	
9	ALDOA	Fructose-bisphosphate aldolase A		+		+			u	d			+	[21]
4	ALDOC	Fructose-bisphosphate aldolase C		+					u	d			+	[22]
3	ALPP	Alkaline phosphatase, placental type precursor	+									+		[23]
10	ANP32A	Acidic leucine-rich nuclear phosphoprotein 32 member A	+	+	+	+	+	+	u	d		+	+	
13	ANP32B	ANP 32 family member B	+	+	+	+	+	+		d	N	+	+	[24]
3	ANP32C	ANP 32 family member C, PP32R1	+	+		+						+	+	
4	ANP32E	ANP 32 family member E	+		+	+			u	d	Orf9c	+	+	
4	ANXA2	Annexin A2	+			+			u	d		+	+	[25]
13	ANXA2P2	Putative annexin A2-like protein, ANX2L2, LPC2B	+			+			u	d			+	[26]
10	ANXA3	Annexin A3				+			u	d			+	[25]
5	ANXA4	Annexin IV				+			u	d			+	[27]
15	ANXA5	Annexin A5	+	+		+			u	d	Orf3		+	[28]
33	ANXA6	Annexin VI	+	+	+	+	+		u	d			+	[29]
2	AP1B1	AP-1 complex subunit beta-1	+										+	
8	AP3B1	AP-3 complex subunit beta-1	+			+			u		E	+		
2	AP3B2	AP-3 complex subunit beta-2	+			+						+		[30]
8	AP3D1	AP-3 complex subunit delta-1	+			+			u	d		+		
4	APEH	Acylamino-acid-releasing enzyme		+									+	
4	APEX1	DNA-(apurinic or apyrimidinic site) lyase		+		+			u	d			+	[31]
2	API5	Apoptosis inhibitor 5		+	+					d			+	
3	APOA1	Apolipoprotein A-I	+							d			+	[32]
2	APOD	Apolipoprotein D	+						u	d			+	
8	ARF1	ADP-ribosylation factor	+	+							Nsp6		+	
2	ARHGAP1	Rho-GTPase-activating protein 1	+						u		Orf3a Orf3b Orf6 Orf7a Orf7b Orf8 Orf9c S		+	
6	ARHGDIA	Rho GDP-dissociation inhibitor 1	+	+					u	d			+	
8	ARHGDIB	Rho GDP-dissociation inhibitor 2		+						d			+	[33]
3	ARPC2	Actin-related protein 2/3 complex subunit 2		+						d			+	
7	ASMTL	N-Acetylserotonin O-methyltransferase-like protein			+								+	
2	ASNS	Glutamine-dependent asparagine synthetase		+					u				+	
4	ASPH	Aspartyl/asparaginyl beta-hydroxylase				+			u	d	Orf9c	+		
14	ATIC	Bifunctional purine biosynthesis protein, PURH		+	+	+							+	[34]
2	ATP2A2	Sarcoplasmic/ER calcium ATPase 2				+			u		Nsp4	+		[35]
13	ATP5F1B	ATP synthase subunit beta, mitochondrial, ATP5B	+	+	+	+	+		u	d	Nsp6 Orf9b	+	+	[36]
3	ATXN10	Ataxin-10, Spinocerebellar ataxia type 10 protein		+					u	d			+	
3	BASP1	Brain acid soluble protein 1 (Neuronal axonal membrane protein NAP22)						+	u	d	M Orf3a Orf7b S		+	
3	BCAT1	Branched chain amino acid aminotransferase	+						u				+	
2	BCCIP	BRCA2 and CDKN1A-interacting protein	+										+	
2	BGN	Biglycan	+									+		[37]
3	BRIX1	Ribosome biogenesis protein BRX1 homolog				+						+		
2	BSG	Basigin, CD147	+							d		+		[38]
3	BTF3	Transcription factor BTF3, NACB			+				u	d			+	
2	BZW1	Basic leucine zipper and W2 domain-containing protein 1		+					u				+	
3	BZW2	Basic leucine zipper and W2 domain-containing protein 2	+	+			+				M		+	
7	C1QBP	Complement C1q-binding protein	+	+	+	+	+	+		d		+	+	[39]
7	CALD1	Caldesmon	+							d			+	[40]
8	CALM1	Calmodulin-1	+	+			+	+	u	d			+	[41]
5	CALM2	Calmodulin-2			+					d			+	
2	CALM3	Calmodulin-3			+		+		u				+	[42]
19	CALR	Calreticulin	+	+	+	+	+	+	u	d			+	[43]
2	CALU	Calumenin	+						u	d	E M Nsp6 Nsp7 Orf3a Orf3b Orf6 Orf7a Orf7b Orf9c S		+	[44]
15	CAND1	Cullin-associated NEDD8-dissociated protein 1		+	+		+						+	
7	CANX	Calnexin	+			+		+	u	d	Nsp4 Orf8	+		[45]
9	CAP1	Adenylyl cyclase-associated protein 1	+	+		+			u	d	Orf3		+	
7	CAPN1	Calpain-1 catalytic subunit	+	+		+							+	
5	CAPN2	Calpain-2 catalytic subunit	+					+	u	d	Nsp16		+	[41]
3	CAPNS1	Calpain small subunit 1	+										+	
3	CAPRIN1	Caprin-1		+	+		+			d			+	
3	CAPZA1	F-actin-capping protein subunit alpha-1	+	+			+			d		+	+	[46]
3	CAPZB	F-actin-capping protein subunit beta	+	+		+				d			+	[47]
8	CAVIN1	Caveolae-associated protein 1, PTRF	+						u	d	N S		+	[48]
3	CBX1	Chromobox protein homolog 1	+		+				u				+	[49]
3	CBX3	Chromobox protein homolog 3			+				u	d			+	
3	CCDC6	Coiled-coil domain-containing protein 6	+						u	d			+	[50]
12	CCT2	T-complex protein 1 subunit beta	+	+	+		+			d	Nsp12 Orf8 Orf9b Orf10		+	[51]
12	CCT3	T-complex protein 1 subunit gamma		+	+		+		u		Orf8 Orf10		+	[52]
6	CCT4	T-complex protein 1 subunit delta		+	+		+		u				+	[52]
10	CCT5	T-complex protein 1 subunit epsilon		+	+		+		u	d	Nsp1 Nsp12 Orf8 Orf10		+	[51]
7	CCT6A	T-complex protein 1 subunit zeta		+	+		+		u	d	Nsp1 Nsp12 Orf10		+	[51]
9	CCT7	T-complex protein 1 subunit eta		+	+		+				Orf10		+	[51]
20	CCT8	T-complex protein 1 subunit theta	+	+	+	+	+		u	d	Nsp1 Nsp12 Nsp14 Nsp15		+	[52]
4	CD248	Endosialin	+							d			+	
7	CDC37	Hsp90 co-chaperone Cdc37	+	+	+		+		u	d	Nsp16		+	
3	CDK11A	Cyclin-dependent kinase 11A, CDC2L2				+			u			+		
3	CEBPZ	CCAAT/enhancer-binding protein zeta				+			u			+		
2	CFL1	Cofilin-1, CFL			+				u	d			+	[53]
4	CKAP4	Cytoskeleton-associated protein 4, P63	+						u	d	Nsp2 Orf7b	+		[54]
8	CKB	Creatine kinase B-type							u	d			+	[55]
7	CLIC1	Chloride intracellular channel protein 1	+	+	+	+			u	d	Nsp16		+	[56]
2	CLIC4	Chloride intracellular channel protein 4	+						u	d			+	
51	CLTC	Clathrin heavy chain 1	+	+		+		+	u	d		+	+	[57]
4	CLTCL1	Clathrin heavy chain 2	+	+		+		+				+	+	
4	CLUH	Clustered mitochondria protein homolog (mRNA-binding)		+						d	Nsp7 Nsp16		+	
2	CMPK1	UMP-CMP kinase		+						d			+	
3	CNDP2	Cytosolic non-specific dipeptidase		+					u		Orf3 Orf10		+	
3	CNPY2	Protein canopy homolog	+	+	+					d	Orf3a		+	
13	COL12A1	Collagen type XII alpha-1 chain	+						u	d			+	
45	COL1A1	Collagen type I alpha-1 chain	+						u	d			+	[58]
37	COL1A2	Collagen type I alpha-2 chain	+							d			+	[59]
2	COL2A1	Collagen type II alpha-1 chain	+						u				+	[60]
12	COL3A1	Collagen type III alpha-1 chain	+										+	[61]
3	COL5A1	Collagen type V alpha 1	+						u				+	[62]
6	COL6A1	Collagen type VI alpha-1 chain	+							d	Orf8		+	[63]
4	COL6A2	Collagen type VI alpha-2 chain	+							d			+	
29	COL6A3	Collagen type VI alpha-3 chain	+							d			+	
2	COPA	Coatomer subunit alpha	+			+			u	d		+		[64]
2	COPB1	Coatomer subunit beta	+							d	Nsp7	+		[65]
5	COPB2	Coatomer subunit beta’	+					+	u			+		[66]
2	COPD	Coatomer subunit delta, ARCN1	+							d	Orf3b Orf6		+	
2	COPG1	Coatomer subunit gamma-1						+			E M Nsp4 Nsp6 Orf3b Orf6 Orf7a Orf7b		+	[39]
2	COPZ1	Coatomer subunit zeta-1	+	+						d			+	
12	CORO1A	Coronin-1A		+					u				+	[67]
3	CORO1C	Coronin-1C	+										+	
3	CPNE1	Copine-1		+									+	
4	CPNE3	Copine-3		+		+			u	d			+	
4	CRK	Proto-oncogene c-Crk	+						u	d	Nsp12 Nsp14 Nsp15		+	
5	CRTAP	Cartilage-associated protein, P3H5	+							d		+		
3	CS	Citrate synthase, mitochondrial		+					u	d	E		+	[3]
4	CSK	Tyrosine-protein kinase CSK		+						d			+	[68]
3	CSNK2A1	Casein kinase 2, alpha 1			+								+	
4	CSPG4	Chondroitin sulfate proteoglycan 4	+							d	Orf7b S	+		[69]
4	CTCFL	High mobility group box 1 pseudogene 1, HMGB1P1, HMGB1L1	+										+	[136]
2	CTR9	RNA polymerase-associated protein CTR9 homolog				+			u	d	Orf9c	+		
3	CTSB	Cathepsin B, APP secretase	+						u	d	M Nsp12		+	
2	CTSD	Cathepsin D	+						u	d			+	[70]
2	CUTA	Protein CutA	+						u	d			+	
6	DAP3	28S ribosomal protein S29, mitochondrial, MRPS29				+						+		
6	DARS	Aspartate-tRNA ligase, DARS1		+									+	[71]
2	DBN1	Drebrin 1	+						u	d			+	[72]
4	DCAF1	DDB1- and CUL4-associated factor 1, VPRBP				+			u	d		+		
3	DCK	Deoxycytidine kinase		+					u				+	
3	DCN	Decorin	+							d		+		[73]
2	DCTN1	Dynactin subunit 1, 150 KDa Dynein-associated protein	+							d		+		[74]
5	DCTN2	Dynactin subunit 2	+	+							Orf6		+	
3	DCTPP1	dCTP pyrophosphatase 1		+						d	Orf9b		+	
28	DDB1	DNA damage-binding protein 1	+	+		+	+	+	u	d		+	+	[57]
3	DDX17	ATP-dependent RNA helicase DDX17				+			u	d		+		[53]
7	DDX18	ATP-dependent RNA helicase DDX18				+			u			+		
5	DDX21	Nucleolar RNA helicase 2			+	+			u	d	N	+		[75]
4	DDX27	ATP-dependent RNA helicase DDX27				+			u			+		
3	DDX30	ATP-dependent RNA helicase DHX30				+				d		+		
7	DDX39A	ATP-dependent RNA helicase DDX39A	+	+	+		+		u	d			+	[39]
5	DDX39B	Spliceosome RNA helicase BAT1	+	+	+		+			d			+	
4	DDX5	ATP-dependent RNA helicase, p68		+		+			u	d		+	+	[76]
16	DDX9	ATP-dependent RNA helicase A, DHX9	+	+	+	+		+				+	+	[77]
2	DEK	Protein DEK			+				u	d			+	[53]
12	DHX15	Pre-mRNA-splicing factor ATP-dependent RNA helicase	+	+		+	+			d		+	+	
4	DHX36	ATP-dependent RNA helicase DHX36				+			u			+		
5	DIABLO	Second mitochondria-derived activator of caspase	+	+	+				u	d	Nsp6 Nsp15		+	
4	DKC1	H/ACA ribonucleoprotein complex subunit B	+			+			u	d		+		
4	DLD	Dihydrolipoyl dehydrogenase, mitochondrial		+									+	[79]
2	DLST	Dihydrolipoyllysine-residue succinyltransferase component of 2-oxoglutarate dehydrogenase complex	+							d			+	[80]
2	DNAJB11	DnaJ (Hsp40) homolog subfamily B member 11	+						u				+	[81]
2	DNAJC8	DnaJ homolog subfamily C member 8		+					u				+	
4	DNPH1	2’-deoxynucleoside 5’-phosphate N-hydrolase 1		+					u				+	
6	DPP3	Dipeptidyl-peptidase 3	+	+		+				d			+	
3	DPYSL2	Dihydropyrimidinase-related protein	+						u	d			+	[82]
3	DRG1	Developmentally-regulated GTP-binding protein	+							d			+	
5	DUT	Deoxyuridine 5’-triphosphate nucleotidohydrolase, mitochondrial		+					u	d			+	
5	DYNC1H1	Dynein cytoplasmic 1 heavy chain 1	+									+		
3	DYNC1I2	Dynein cytoplasmic 1 intermediate chain 2	+			+						+		
3	EBP2	Probable rRNA-processing protein, EBNA1BP2				+						+		
4	ECH1	Delta(3,5)-delta(2,4)-dienoyl-CoA isomerase, mitochondrial				+			u	d			+	[83]
2	EEF1A1	Elongation factor 1-alph 1	+	+		+			u	d			+	[84]
4	EEF1A2	Elongation factor 1-alpha 2	+	+		+			u		Orf3		+	[85]
2	EEF1B2	Elongation factor 1-beta 2	+	+	+		+			d			+	
5	EEF1D	Elongation factor 1-delta	+	+	+					d			+	[86]
10	EEF1G	Elongation factor 1-gamma	+	+	+	+	+		u	d			+	
17	EEF2	Elongation factor 2	+	+	+	+			u	d		+	+	[87]
16	EFTUD2	116 kDa U5 snRNP component, SNRP116	+	+	+	+		+		d		+	+	[88]
4	EHD2	EH domain-containing protein 2	+						u	d			+	
3	EIF2A	Eukaryotic translation initiation factor 2 subunit 1, EIF2S1	+	+	+	+							+	[89]
10	EIF3A	Eukaryotic translation initiation factor 3 subunit A	+	+		+			u	d	Nsp1	+	+	[90]
9	EIF3B	Eukaryotic translation initiation factor 3 subunit B	+	+		+			u	d	Nsp1	+	+	[39]
2	EIF3C	Eukaryotic translation initiation factor 3 subunit C		+	+					d	Nsp1		+	[91]
3	EIF3CL	Eukaryotic translation initiation factor 3 subunit C-like protein	+			+				d		+		
5	EIF3E	Eukaryotic translation initiation factor 3 subunit E	+	+	+	+			u	d	Nsp1	+	+	[92]
4	EIF3F	Eukaryotic translation initiation factor 3 subunit F	+	+					u	d	Nsp1	+	+	[93]
2	EIF3G	Eukaryotic translation initiation factor 3 subunit G	+								Nsp1		+	[93]
2	EIF3I	Eukaryotic translation initiation factor 3 subunit I		+						d	Nsp1		+	[91]
10	EIF3L	EIF3, subunit E interacting protein	+	+	+	+				d	Nsp1	+	+	[39]
19	EIF4A1	Eukaryotic initiation factor 4A-1, DDX2A	+	+		+	+		u	d			+	
8	EIF4A3	Eukaryotic initiation factor 4A-III, DDX48	+	+		+						+	+	[94]
4	EIF4G1	Eukaryotic translation initiation factor 4 gamma 1	+		+				u	d			+	[93]
2	EIF4G2	Eukaryotic translation initiation factor 4 gamma 2	+							d	Nsp1		+	[93]
2	EIF5	Eukaryotic translation initiation factor 5		+					u	d			+	[95]
5	EIF5A	Eukaryotic translation initiation factor 5A-1	+	+		+			u	d			+	[95]
2	EIF5A2	Eukaryotic translation initiation factor 5A-2	+		+		+			d			+	[95]
2	EIF5B	Eukaryotic translation initiation factor 5b (eif-5b) (translation initiation factor if-2)			+				u				+	
3	EIF6	Eukaryotic translation initiation factor 6	+	+	+				u				+	
4	ELAVL1	ELAV-like protein	+	+						d			+	[96]
2	ELOB	Transcription elongation factor B, TCEB2	+						u	d	Nsp16 Orf10		+	
2	EMG1	Ribosomal RNA small subunit methyltransferase NEP1				+			u	d			+	
12	ENO1	Alpha-enolase	+		+	+		+	u	d			+	[97]
7	ENO2	Gamma-enolase	+	+					u	d			+	[98]
2	ENOPH1	Enolase-phosphatase E1	+						u				+	
6	EPHX1	Epoxide hydrolase				+				d		+		[99]
4	ERO1A	Endoplasmic oxidoreductin-1-like protein, ERO1L		+				+		d	Orf3a		+	
6	ERP44	Endoplasmic reticulum resident protein ERp44	+								Orf8		+	[101]
4	ESYT1	Extended synaptotagmin-1, FAM62A	+			+					E M Nsp3 Nsp4 Nsp6 Orf3a Orf6 Orf7a Orf7b Orf8 Orf9c S	+		[102]
4	ETF1	Eukaryotic peptide chain release factor subunit 1		+					u				+	
2	EWSR1	EWS RNA-binding protein	+						u	d			+	
14	EZR	Ezrin		+		+			u	d	S		+	[103]
2	FAF1	FAS-associated factor 1	+						u				+	
3	FARSB	Phenylalanine-tRNA ligase beta subunit		+									+	[104]
19	FASN	Fatty acid synthase	+	+	+		+		u	d			+	[105]
3	FBLN1	Fibulin 1	+						u	d			+	[106]
2	FDPS	Farnesyl pyrophosphate synthetase like-4 protein		+						d			+	
2	FEN1	Flap endonuclease 1		+					u	d			+	
2	FERMT3	Fermitin family homolog 3		+					u				+	
8	FKBP10	FK506-binding protein 10	+								Orf8		+	
11	FKBP4	Peptidyl-prolyl cis-trans isomerase FKBP4, FKBP-52		+		+					Nsp12		+	[107]
2	FKBP5	Peptidyl-prolyl cis-trans isomerase FKBP5 (FK506-binding protein)		+					u				+	
4	FKBP9	FK506-binding protein 9	+							d			+	
43	FLNA	Filamin-A	+			+		+	u	d		+	+	[108]
25	FLNB	Filamin-B	+			+		+	u				+	[57]
24	FLNC	Filamin-C	+			+			u	d		+	+	[109]
23	FN1	Fibronectin	+						u	d			+	[110]
3	FSTL1	Follistatin-related protein	+						u	d			+	[111]
2	FTH1	Ferritin heavy chain	+			+			u	d			+	[111]
2	FUBP1	Far upstream element-binding protein 1		+					u	d			+	[112]
10	G6PD	Glucose-6-phosphate 1-dehydrogenase	+	+		+			u	d		+	+	[44]
15	GANAB	Neutral alpha-glucosidase AB	+	+		+				d	Orf6 Orf8 Orf9c		+	[113]
6	GAPDH	Glyceraldehyde-3-phosphate dehydrogenase	+	+	+	+			u	d	Orf8	+	+	[114]
2	GAR1	H/ACA ribonucleoprotein complex subunit 1	+			+						+		
4	GARS	Glycine-tRNA ligase, GARS1		+					u				+	[115]
2	GART	Trifunctional purine biosynthetic protein adenosine-3		+						d	Nsp15		+	
2	GBE1	1,4-alpha-glucan-branching enzyme		+		+			u				+	
4	GCLC	Glutamate-cysteine ligase catalytic subunit				+					Orf3		+	
8	GDI1	Rab GDP dissociation inhibitor alpha	+	+		+			u	d			+	[116]
10	GDI2	Rab GDP dissociation inhibitor beta	+	+		+			u	d	Nsp12 Orf9b		+	[117]
2	GGCT	Gamma-glutamylcyclotransferase, cytochrome c-releasing factor 21		+					u				+	
3	GLO1	Lactoylglutathione lyase		+		+				d	Orf3		+	[118]
3	GLRX3	Glutaredoxin 3, Thioredoxin-like 2	+	+						d			+	[119]
10	GLUD1	Glutamate dehydrogenase 1, mitochondrial		+									+	[120]
2	GMFB	Glia maturation factor, beta	+	+					u				+	
2	GPALPP1	Lipopolysaccharide-specific response protein 7			+								+	
5	GPC1	Glypican-1	+			+				d		+		
2	GPI	Glucose-6-phosphate isomerase				+			u	d	E Nsp6 Orf3 Orf3b Orf6		+	[121]
4	GRWD1	Glutamate-rich WD repeat-containing protein 1				+						+		
16	GSN	Gelsolin	+						u	d			+	[16]
3	GSPT1	Eukaryotic peptide chain release factor GTP-binding subunit ERF3A		+									+	
3	GSS	Glutathione synthetase		+						d			+	
6	GSTP1	Glutathione S-transferase				+			u	d			+	[122]
4	GTF2I	General transcription factor II-I	+	+					u	d			+	[25]
3	H1-1	Histone H1.1, H1F1, HIST1H1A, H1FNT			+		+		u	d		+	+	[123]
2	H1F0	Histone H1.0, H1FV, H1-0						+	u	d	Nsp3 Nsp8 Orf3b Orf10		+	
3	H2AFV	Histone H2A.V, H2AZ2	+	+		+		+	u	d		+	+	[127]
11	H2AFY	Core histone macro-H2A.1, MACROH2A1				+		+	u			+		[128]
4	H2AFY2	Cor2 histone macro-H2A.2, MACROH2A2	+	+		+			u			+		[128]
4	HADHA	Trifunctional enzyme subunit alpha, mitochondrial				+						+		
3	HARS	Histidyl-tRNA synthetase, cytoplasmic	+	+		+							+	[41]
5	HDGF	Hepatoma-derived growth factor, HMG1L2	+	+	+		+	+	u	d			+	[134]
2	HDLBP	Vigilin, High density lipoprotein binding protein	+						u	d	N Nsp2		+	
4	HEATR1	HEAT repeat-containing protein 1				+			u	d		+		
2	HEBP2	Heme-binding protein 2	+						u				+	
5	HEXB	Beta-hexosaminidase subunit beta	+							d			+	
6	HIST1H1B	Histone H1.5, H1F5, H1-5	+		+	+	+	+	u	d		+	+	[124]
6	HIST1H1C	Histone H1.2, H1F2, H1-2	+	+	+	+	+	+	u	d	Nsp8	+	+	[124]
4	HIST1H2A A	Histone H2A type 1-A, H2AFR, H2AC1			+	+	+					+	+	[125]
2	HIST1H2AB	Histone H2A type 1-B/E, H2AFM, H2AC4		+						d		+		[126]
5	HIST1H2BA	Histone H2B type 1-A, H2BC1			+		+					+	+	[123]
5	HIST1H2BB	Histone H2B type 1-B, H2BFF, H2BC3			+		+					+		[131]
2	HIST1H2BL	Histone H2B type 1-L, H2BFC, H2BC13	+	+		+			u	d		+		[129]
12	HIST2H2BE	Histone H2B type 2-E, H2BC21	+	+		+		+	u	d		+		[130]
5	HIST2H3A	Histone H3.2, H3C15	+	+		+		+	u	d		+	+	[132]
4	HIST3H3	Histone H3.1t, H3FT, H3-4			+		+					+	+	[123]
14	HIST4H4	Histone H4, H4C1	+	+	+	+	+	+	u	d		+	+	[133]
10	HMGB1	High mobility group protein 1		+	+		+			d			+	[135]
3	HMGCS1	Hydroxymethylglutaryl-CoA synthase, cytoplasmic		+	+		+		u	d			+	
2	HMGN1	Non-histone chromosomal protein HMG14			+				u				+	
4	HNRNPA1	Heterogeneous nuclear ribonucleoprotein A1	+	+	+		+	+	u	d			+	[137]
8	HNRNPA2 B1	hnRNP A2/B1	+	+	+	+		+	u	d			+	[138]
2	HNRNPA3	hnRNP A3	+	+				+	u	d			+	[139]
2	HNRNPAB	hnRNP A/B		+						d			+	[139]
3	HNRNPC	hnRNP C1/C2	+	+	+	+			u	d		+	+	[140]
7	HNRNPCL1	hnRNP C-like 1	+	+	+	+	+	+				+	+	[141]
5	HNRNPD	hnRNP D, AUF1	+	+	+								+	[142]
3	HNRNPDL	hnRNP D-like	+	+					u	d			+	[143]
5	HNRNPF	hnRNP F	+	+		+				d		+	+	[144]
2	HNRNPH1	hnRNP H1	+	+	+	+			u	d			+	
2	HNRNPH3	hnRNP H3	+						u	d			+	[145]
9	HNRNPK	hnRNP K	+	+	+	+	+		u				+	[146]
3	HNRNPM	hnRNP M				+			u	d		+		
6	HNRNPQ	hnRNP Q, SYNCRIP	+	+	+	+	+	+		d			+	
7	HNRNPR	hnRNP R	+	+	+	+		+	u	d			+	[147]
5	HNRNPU	hnRNP U (scaffold attachment factor A)	+	+	+	+	+	+	u	d		+	+	[148]
6	HNRNPUL1	hnRNP U-like protein 1	+			+			u	d		+		
4	HNRNPUL 2	hnRNP U-like protein 2				+			u	d		+		
6	HPRT1	Hypoxanthine-guanine phosphoribosyltransferase		+									+	
2	HSP70B	Putative heat shock 70 kDa protein, HSPA7				+			u	d		+		
38	HSP90AA1	Heat shock protein 90-alpha	+	+	+	+	+	+	u	d		+	+	[149]
6	HSP90AA2	Heat shock protein 90-alpha A2	+	+		+		+	u			+	+	[150]
16	HSP90AB1	Heat shock protein HSP 90-beta	+	+	+	+	+	+	u	d	Nsp12		+	[151]
31	HSP90B1	Endoplasmin, GRP94	+	+	+	+	+	+	u	d	Orf3a Orf9c	+	+	[152]
7	HSPA1A	Heat shock 70 kDa protein 1A	+			+			u	d	N Orf9b		+	
4	HSPA1L	Heat shock 70 kDa protein 1-like, HSP70T	+			+							+	[153]
2	HSPA2	Heat shock 70 kda protein 2			+				u		Nsp3	+		
14	HSPA4	Heat shock 70 kDa protein 4	+	+	+		+		u	d			+	[154]
35	HSPA5	Endoplasmic reticulum chaperone BiP, GRP78	+	+	+	+	+	+	u	d	E M Nsp2 Nsp4 Nsp6 Orf3a Orf7a Orf7b S		+	[155]
27	HSPA8	Heat shock cognate 71 kDa protein	+	+	+	+		+	u	d	Nsp2 Nsp12		+	[156]
25	HSPA9	Stress-70 protein, mitochondrial (GRP75)	+	+	+	+		+	u	d	N		+	[156]
7	HSPB1	Heat shock protein beta-1	+						u	d			+	[157]
2	HSPBP1	Hsp70-binding protein 1				+			u	d	S		+	
30	HSPD1	60 kDa heat shock protein, mitochondrial	+	+	+		+		u	d			+	[158]
3	HSPG2	Basement membrane heparan sulfate proteoglycan	+			+			u	d		+		[159]
13	HSPH1	Heat shock protein 105 kDa		+	+		+		u				+	[160]
4	HTATSF1	HIV Tat-specific factor 1	+			+				d		+		
7	HYOU1	Hypoxia up-regulated protein	+		+		+		u		Nsp4 Orf3a Orf8		+	[161]
4	IDE	Insulin-degrading enzyme		+		+					Nsp4 Nsp12 Nsp14 Nsp15 Nsp16 Orf3b		+	
2	IDH3A	Isocitrate dehydrogenase [NAD] subunit alpha, mitochondrial		+									+	
2	IGBP1	Immunoglobulin-binding protein 1	+						u	d			+	
2	IL18	Interleukin-18				+			u	d			+	[162]
7	ILF2	Interleukin enhancer-binding factor 2	+	+	+	+			u			+	+	[163]
6	ILF3	Interleukin enhancer-binding factor 3	+		+				u				+	[163]
2	IMPDH2	Inosine-5’-monophosphate dehydrogenase 2 (imp dehydrogenase 2) (impdh-ii)			+					d	Nsp14		+	
7	IPO5	Importin-5, KPNB3, RANBP5		+	+		+						+	[164]
3	IPO7	Importin-7, RANBP7		+							Nsp6 Orf9c		+	[165]
13	IQGAP1	Ras GTPase-activating-like protein IQGAP1	+	+		+		+	u			+	+	[166]
2	IRGQ	Immunity-related GTPase family Q protein	+						u	d			+	
4	ITGB1	Integrin beta-1	+			+			u	d	Nsp4 Of3b Orf6 Orf8 Orf9c	+		[167]
2	IWS1	Protein IWS1 homolog				+			u	d		+		
4	KARS	Lysyl-tRNA synthetase	+	+		+					Nsp7	+	+	[100]
3	KHSRP	Far upstream element-binding protein 2 (KH-type splicing regulatory protein), FUBP2		+					u	d			+	[53]
2	KPNA2	Importin subunit alpha-1		+						d	Orf6		+	
2	KPNA3	Importin subunit alpha-4	+		+							+	+	
11	KPNB1	Importin subunit beta-1	+	+	+	+	+	+				+	+	[164]
2	KRR1	KRR1 small subunit processome component homolog, HIV-1 Rev-binding protein				+				d		+		[168]
10	KTN1	Kinectin	+						u		Orf6		+	[169]
2	KYNU	Kynureninase				+			u		Orf3		+	
7	LAMB1	Laminin subunit beta-1	+							d			+	[170]
5	LAMC1	Laminin subunit gamma-1	+						u	d			+	[171]
2	LAMP2	Lysosome-associated membrane glycoprotein 2				+			u	d			+	[172]
2	LARS	Leucyl-tRNA synthetase, cytoplasmic				+						+		[100]
8	LDHA	L-lactate dehydrogenase A chain		+	+	+			u	d	Nsp12		+	[173]
10	LDHB	L-lactate dehydrogenase B chain		+	+	+			u	d	Nsp12 Nsp7		+	[174]
2	LEO1	RNA polymerase-associated protein LEO1				+			u			+		
5	LGALS1	Galectin-1	+						u	d			+	[175]
23	LMNA	Prelamin-A/C	+			+		+	u	d	Nsp13 Orf3b Orf8 Orf10		+	[176]
8	LMNB1	Lamin-B1	+		+		+		u	d		+	+	[177]
7	LMNB2	Lamin-B2	+			+			u	d		+		[178]
16	LRPPRC	Leucine-rich PPR motif-containing protein	+	+	+	+		+		d			+	[179]
2	LSM2	U6 snRNA-associated Sm-like protein LSm2	+						u				+	
2	LSM6	U6 snRNA-associated Sm-like protein LSm6	+						u				+	
2	LSM8	U6 snRNA-associated Sm-like protein LSm8					+						+	
2	MAGOHB	Protein mago nashi homolog	+						u	d			+	
3	MANBA	Beta-mannosidase	+							d			+	
3	MAP1B	Microtubule-associated protein 1B	+			+			u	d		+	+	[180]
6	MAPRE1	Microtubule-associated protein RP/EB family member	+		+		+				Orf3		+	
2	MARCKS	Myristoylated alanine-rich c-kinase substrate (marcks) (protein kinase c substrate, 80 kda protein, light chain) (pkcsl)			+				u	d	M Nsp4 Nsp6 Orf3a Orf3b Orf7b S		+	
2	MARS	Methionine-tRNA ligase, MARS1				+				d		+		[39]
9	MCM2	DNA replication licensing factor MCM2		+	+		+	+		d			+	[181]
7	MCM3	DNA replication licensing factor MCM3		+	+		+	+	u	d			+	[39]
5	MCM4	DNA replication licensing factor MCM4		+	+		+		u	d			+	[181]
3	MCM5	DNA replication licensing factor MCM5			+		+		u	d			+	[181]
9	MCM6	DNA replication licensing factor MCM6		+	+		+	+	u	d			+	[39]
2	MDH1	Malate dehydrogenase, cytoplasmic			+					d	E Orf3		+	
3	MDH2	Malate dehydrogenase, mitochondrial				+			u	d			+	[25]
2	ME2	NAD-dependent malic enzyme, mitochondrial		+					u	d	Nsp15		+	
10	MOV10	Putative helicase, Moloney leukemia virus 10 protein	+			+			u	d	E M N Nsp3 Nsp4 Nsp6 Orf3a Orf7a Orf7b Orf8 Orf9c S	+		
5	MRPL1	39S ribosomal protein L1, mitochondrial				+						+		
3	MRPL13	39S ribosomal protein L13, mitochondrial				+				d		+		
2	MRPL15	39S ribosomal protein L15, mitochondrial				+			u	d		+		
2	MRPL17	39S ribosomal protein L17, mitochondrial				+				d		+		
2	MRPL18	39S ribosomal protein L18, mitochondrial				+				d		+		
4	MRPL19	39S ribosomal protein L19, mitochondrial				+				d	Orf8	+		
2	MRPL2	39S ribosomal protein L2, mitochondrial				+				d	Nsp6	+		
2	MRPL23	39S ribosomal protein L23, mitochondrial				+				d		+		
5	MRPL37	39S ribosomal protein L37, mitochondrial				+			u	d		+		
5	MRPL38	39S ribosomal protein L38, mitochondrial				+				d		+		
2	MRPL39	39S ribosomal protein L39, mitochondrial				+		+		d		+	+	
3	MRPL45	39S ribosomal protein L45, mitochondrial				+				d		+		
2	MRPL49	39S ribosomal protein L49, mitochondrial				+				d		+		
4	MRPS22	28S ribosomal protein S22, mitochondrial				+						+		
4	MRPS23	28S ribosomal protein S23, mitochondrial				+						+		
6	MRPS27	28S ribosomal protein S27, mitochondrial				+					Nsp8	+		
2	MRPS28	28S ribosomal protein S28, mitochondrial, MRPS35				+						+		
2	MRPS30	28S ribosomal protein S30, mitochondrial				+				d		+		
2	MRPS34	28S ribosomal protein S34, mitochondrial				+				d		+		
3	MRPS9	28S ribosomal protein S9, mitochondrial				+						+		
6	MSN	Moesin	+	+		+			u		Nsp6 Orf3 S		+	[182]
21	MVP	Major vault protein	+			+			u	d		+		[183]
4	MXRA5	Matrix-remodeling-associated protein 5	+							d		+		[183]
16	MYBBP1A	Myb-binding protein 1A			+	+		+	u	d		+	+	
2	MYG1	UPF0160 protein MYG1, mitochondrial, C12orf10		+		+							+	
2	MYH10	Myosin-10	+						u	d	Nsp9	+		[184]
43	MYH9	Myosin-9	+		+	+		+	u	d		+	+	[184]
3	MYL6	Myosin light chain 6	+				+		u				+	[185]
4	MYLK	Myosin light chain kinase, smooth muscle	+						u	d			+	
3	MYO1C	Unconventional myosin-Ic, MYO1E	+			+			u	d		+		[186]
4	MZB1	Marginal zone B- and B1-cell-specifc protein (Proapoptotic caspase adapter protein, plasma cell-induced resident protein)		+					u				+	
3	NAA15	N-alpha-acetyltransferase 15, NatA auxiliary subunit (NMDA receptor-regulated protein, NARG1)		+						d			+	
2	NAA25	N-alpha-acetyltransferase 25, NatB auxiliary subunit (TPR repeat-containing protein C12orf30)		+									+	
4	NACA	Nascent polypeptide associated complex subunit alpha	+	+	+		+		u	d			+	[187]
7	NAP1L1	Nucleosome assembly protein 1-like 1	+	+	+	+			u	d		+	+	
7	NAP1L4	Nucleosome assembly protein 1-like 4	+	+	+				u	d		+	+	
5	NARS	Asparagine-tRNA ligase, cytoplasmic, NARS1		+						d			+	[188]
6	NASP	Nuclear autoantigenic sperm protein	+	+	+		+		u	d			+	[189]
23	NCL	Nucleolin	+	+	+	+	+	+	u	d		+	+	[190]
2	NES	Nestin	+						u	d			+	
2	NEU1	Sialidase-1	+						u	d	Orf8		+	[191]
3	NEXN	Nexilin F-actin binding protein	+						u	d			+	
2	NFU1	HIRA interacting protein 5	+										+	
8	NME1	Nucleoside diphosphate kinase A, RMRP	+	+		+			u	d			+	[192]
3	NME2	Nucleoside diphosphate kinase 2, NM23				+			u	d			+	[193]
2	NMT1	Glycylpeptide N-tetradecanoyltransferase 1	+										+	[194]
2	NMT2	Glycylpeptide N-tetradecanoyltransferase 2	+							d			+	
2	NOC2L	Nucleolar complex protein 2 homolog				+				d		+		
7	NOLC1	Nucleolar phosphoprotein p130 (nucleolar and coiled-body phosphoprotein 1)			+				u	d			+	
9	NOP2	Probable 28S rRNA (cytosine(4447)-C(5)-methyltransferase				+			u			+		
15	NPEPPS	Puromycin-sensitive aminopeptidase, metalloproteinase MP100	+	+		+							+	
7	NPM1	Nucleophosmin (nucleolar phosphoprotein, numatrin)	+	+	+	+	+	+	u	d	Orf9c	+	+	[195]
2	NRCAM	Neuronal cell adhesion molecule				+			u	d			+	[196]
3	NSFL1C	NSFL1 cofactor p47		+					u				+	
8	NUDC	Nuclear distribution C, Dynein complex regulator	+	+	+					d	Nsp12		+	
4	NUDT21	Cleavage and polyadenylation specificity factor 5	+	+		+				d			+	
2	NUDT3	Diphosphoinositol polyphosphate phosphohydrolase		+									+	
4	NUDT5	Nudix hydrolase 5	+	+	+		+			d			+	
3	NUMA1	Nuclear mitotic apparatus protein 1	+						u	d			+	[197]
2	OLA1	Obg-like ATPase 1				+			u				+	
2	OTUB1	Ubiquitin thioesterase protein OTUB1		+									+	
5	P3H1	Basement membrane chondroitin sulfate proteoglycan	+						u				+	
2	P3H3	Prolyl 3-hydroxylase 3, LEPREL2	+							d		+		
2	P3H4	ER protein SC65, nucleolar autoantigen No55	+								M	+		[198]
2	P4HA2	Prolyl 4-hydroxylase subunit alpha-2	+							d			+	
18	P4HB	Protein disulfide-isomerase	+	+	+	+	+	+	u	d	Nsp7 Orf3b		+	[199]
14	PA2G4	Proliferation-associated protein 2G4	+	+					u	d			+	
22	PABPC1	Poly(A)-binding protein 1	+	+		+				d	N	+		[200]
9	PABPC3	Poly(A)-binding protein 3			+		+	+		d		+	+	
16	PABPC4	Poly(A)-binding protein 4, APP1	+		+	+		+		d	N	+	+	[201]
4	PAF1	RNA polymerase II-associated factor 1 homolog				+				d		+		
2	PAFAH1B2	Platelet-activating factor acetylhydrolase IB subunit beta		+		+			u	d			+	
3	PAFAH1B3	Platelet-activating factor acetylhydrolase IB subunit gamma		+		+			u		Nsp12 Nsp5 Orf3b		+	
6	PAICS	Multifunctional protein ADE2		+						d			+	
2	PARP1	Poly [ADP-ribose] polymerase 1		+					u	d			+	
3	PARVA	Alpha-parvin	+						u				+	
8	PCNA	Proliferating cell nuclear antigen	+	+	+	+	+	+	u	d			+	[202]
2	PDCD10	Programmed cell death protein 10		+									+	
21	PDIA3	Protein disulfide-isomerase A3	+	+		+			u	d	M Orf3a Orf3b Orf8 Orf9c		+	[203]
34	PDIA4	Protein disulfide-isomerase A4	+	+	+	+	+	+	u	d	Nsp16 Nsp7 Orf3b		+	[204]
10	PDIA6	Protein disulfide-isomerase A6	+	+	+	+	+	+	u	d			+	[205]
6	PELP1	Proline-, glutamic acid-, leucine-rich protein 1				+				d		+		
2	PES1	Pescadillo homolog				+				d		+		
7	PFAS	Formylglycinamide ribonucleotide amidotransferase		+		+					Nsp7 Nsp12 Nsp15 Nsp16		+	
3	PFDN2	Prefoldin subunit 2	+	+					u		Nsp12 Nsp15 Orf10		+	[206]
4	PFDN3	Prefoldin subunit 3, von hippel-lindau-binding protein 1, VBP1	+	+	+		+			d	Nsp12 Nsp15		+	
2	PFKP	ATP-dependent 6-phofructokinase, platelet type				+			u	d	Orf7a		+	[207]
9	PFN1	Profilin-1	+	+	+				u	d			+	[208]
2	PFN2	Profilin-2	+						u				+	[181]
4	PGAM1	Phosphoglycerate mutase 1		+					u	d			+	[209]
4	PGAM2	Phosphoglycerate mutase 2		+		+							+	
9	PGD	6-phosphogluconate dehydrogenase, decarboxylating				+			u	d			+	
3	PGLS	6-phosphogluconolactonase		+					u				+	
3	PHGDH	D-3-phosphoglycerate dehydrogenase		+					u	d			+	[210]
2	PLA2G4A	Cytosolic phospholipase a2			+								+	
10	PLCG2	1-phosphatidylinositol-4,5-bisphosphate phosphodiesterase gamma-2		+					u				+	
2	PLD3	Phospholipase D3, 5’–3’ exonuclease PLD3				+			u	d	Nsp2 Nsp3 Nsp5 Orf6 Orf7b Orf8 Orf10		+	
91	PLEC	Plectin-1, PLEC1	+			+			u	d		+	+	[211]
5	PLOD1	Procollagen-lysine, 2-oxoglutarate 5-dioxygenase 1	+							d			+	
5	PLOD3	Multifunctional procollagen lysine hydroxylase and glycosyltransferase LH3	+										+	
2	PLS1	Plastin-1				+				d			+	
30	PLS2	Plastin-2, LCP1	+	+	+	+	+		u	d			+	[212]
6	PLS3	Plastin-3	+			+			u	d			+	
2	PMPCB	Mitochondrial-processing peptidase subunit beta		+						d	M		+	
2	POP1	Ribonucleases P/MRP protein subunit POP1				+			u			+		[213]
3	POR	NADPH--cytochrome P450 reductase				+			u	d	Nsp2 Orf6	+		
8	PPA1	Inorganic pyrophosphatase		+		+			u		Orf3		+	[214]
3	PPAT	Amidophosphoribosyltransferase		+						d			+	
10	PPIB	Peptidyl-prolyl cis-trans isomerase	+	+		+			u	d	Orf8		+	[215]
3	PPM1G	Protein phosphatase 1G (PPM1C)			+			+			Orf9b		+	
2	PPP1R7	Protein phosphatase 1 regulatory subunit 7 (subunit 22)		+			+		u				+	
7	PPP2R1A	Serine/threonine-protein phosphatase 2A 65 kDa regulatory subunit A alpha isoform		+	+		+			d			+	
6	PRDX1	Peroxiredoxin-1		+		+			u	d			+	[216]
5	PRDX3	Thioredoxin-dependent peroxide reductase	+	+		+			u	d			+	[217]
3	PRDX4	Peroxiredoxin-4	+			+			u	d	Orf3a		+	[218]
2	PRKAR2A	Protein kinase CAMP-dependent type II regulatory alpha	+						u		Nsp1 Orf9b		+	[48]
2	PRKCDBP	Protein kinase C delta-binding protein	+										+	
11	PRKCSH	Protein kinase C substrate 80K-H	+	+	+		+	+		d	Nsp6 Orf3 Orf3a S		+	
17	PRKDC	DNA-dependent protein kinase catalytic subunit (DNA-PKcs)	+		+	+		+	u	d	M Nsp4	+	+	[219]
5	PRMT1	Protein arginine N-methyltransferase 1 (Histone-arginine N-methyltransferase)	+	+	+	+	+	+		d			+	[220]
24	PRPF8	Pre-mRNA-processing-splicing factor 8 (U5 snRNP-specific protein 220 kDa)	+			+		+	u	d		+	+	[57]
2	PRPSAP2	Phosphoribosyl pyrophosphate synthetase-associated protein 2		+					u				+	
2	PSAP	Proactivator polypeptide, Prosaposin	+						u	d			+	
6	PSAT1	Phosphoserine aminotransferase 1				+			u	d	Orf3 Orf7a		+	
3	PSMA1	Proteasome subunit alpha type-1		+	+		+			d	Orf3b		+	[25]
2	PSMA2	Proteasome subunit alpha type-2		+			+	+		d			+	
6	PSMA3	Proteasome subunit alpha type-3, C8	+	+			+		u	d	Nsp2 Nsp4 Nsp7 Nsp10 Nsp12		+	[221]
5	PSMA4	Proteasome subunit alpha type-4, C9	+	+					u				+	[25]
5	PSMA5	Proteasome subunit alpha type-5	+	+	+		+	+	u		Orf3b		+	[222]
8	PSMA6	Proteasome subunit alpha type-6	+	+					u	d	Orf3b		+	
6	PSMA7	Proteasome subunit alpha type-7	+	+	+		+	+	u	d			+	[223]
3	PSMA8	Proteasome subunit alpha type 7-like			+		+						+	[223]
5	PSMB1	Proteasome subunit beta type-1	+	+									+	[224]
3	PSMB3	Proteasome subunit beta type-3	+	+			+			d	Orf3b		+	[221]
7	PSMB4	Proteasome subunit beta type-4	+	+	+		+				Orf3b		+	[25]
3	PSMB6	Proteasome subunit beta type-6	+	+	+					d	Orf3b		+	
5	PSMB7	Proteasome subunit beta type-7	+	+			+			d			+	[221]
3	PSMB8	Proteasome subunit beta type-8		+					u	d			+	
4	PSMB9	Proteasome subunit beta type-9		+					u	d			+	
2	PSMC1	26s Proteasome regulatory subunit 4					+			d	Orf6		+	
2	PSMC3	26S protease regulatory subunit 6A		+						d	Orf6		+	
5	PSMD1	26S proteasome non-ATPase regulatory subunit 1	+	+		+			u		Nsp7 Orf6 Orf8	+	+	
9	PSMD11	Proteasome 26S non-ATPase regulatory subunit 11		+	+				u				+	
3	PSMD12	26S proteasome non-ATPase regulatory subunit 12	+	+						d		+	+	
3	PSMD13	Proteasome 26S non-ATPase subunit 13	+					+		d			+	[225]
2	PSMD14	26S proteasome non-ATPase regulatory subunit 14		+									+	
8	PSMD3	26S proteasome non-ATPase regulatory subunit 3		+						d			+	
9	PSMD6	26S proteasome non-ATPase regulatory subunit 6	+	+		+		+					+	
2	PSMD7	26S proteasome non-ATPase regulatory subunit 7	+						u				+	
11	PSME1	Proteasome activator complex subunit 1		+					u		Nsp15		+	
8	PSME2	Proteasome activator complex subunit 2		+					u				+	
4	PSME3	Proteasome activator complex subunit 3		+	+		+			d	Nsp16		+	[226]
2	PSPH	Phosphoserine phosphatase		+									+	
6	PTBP1	Polypyrimidine tract-binding protein, hnRNP I	+	+					u	d			+	[227]
2	PTBP3	Polypyrimidine tract-binding protein, ROD1	+	+					u	d			+	[227]
16	PTCD3	Pentatricopeptide repeat-containing protein 3, mitochondrial, MRPS39	+			+						+		
2	PTGES3	Prostaglandin E synthase 3 (telomerase-binding protein p23) (hsp90 co-chaperone) (progesterone rec)		+	+		+			d			+	
4	PTMA	Prothymosin alpha			+		+	+	u	d			+	[228]
2	PTPN6	Tyrosine-protein phosphatase non-receptor type 6		+					u	d			+	
2	PUF60	Poly(U)-binding-splicing factor PUF60	+						u				+	[229]
18	PUM1	Pumilio homolog 1				+				d		+		
3	PURA	Transcriptional activator protein Pur-alpha				+			u	d		+		
2	PUS1	tRNA pseudouridine synthase A		+									+	
2	PZP	Pregnancy zone protein, alpha-2macroglobulin like	+							d			+	[230]
4	QARS	Bifunctional glutamate/proline-tRNA ligase, EPRS, EPRS1	+	+		+			u			+	+	[100]
3	RAB1A	Ras-related protein Rab-1A	+			+				d	Nsp7 Orf3 Orf7b	+		
5	RAB7A	Ras-related protein Rab-7a	+	+					u	d	Nsp7 Orf3 Orf7b		+	
3	RAD23A	UV excision repair protein RAD23 homolog A	+	+						d			+	[231]
5	RAD23B	UV excision repair protein RAD23 homolog B	+						u	d	Orf3a Orf3b Orf7a Orf9c		+	[231]
6	RALY	RNA binding protein, autoantigen p542	+			+			u	d	Orf9c	+		[232]
3	RANBP1	Ran-specific GTPase-activating protein		+					u	d			+	
2	RANBP6	Ran-binding protein 6		+						d	Orf7a		+	
2	RANGAP1	Ran GTPase-activating protein 1			+	+				d		+		[165]
3	RARS	Arginyl-tRNA synthetase, cytoplasmic, RARS1				+			u			+		[39]
5	RBBP4	Chromosome assembly factor 1 subunit C	+			+				d		+		[233]
3	RBBP7	Histone-binding protein rbbp7			+		+		u	d			+	
2	RBM3	Putative RNA-binding protein 3	+						u	d	Orf8		+	
3	RBM8A	RNA-binding protein 8A				+			u			+		
2	RBMXL2	RNA-binding motif protein X-linked-like-2	+										+	
2	RCN3	Reticulocalbin-3	+										+	
8	RDX	Radixin	+	+	+	+			u	d	Nsp13		+	[234]
2	RMI2	RecQ-mediated genome instability protein 2			+							+		
3	RNPEP	Arginine aminopeptidase, APB				+					Orf3		+	
2	RNPS1	RNA-binding protein with serine-rich domain 1				+			u	d		+		
4	RO52	E3 ubiquitin-protein ligase TRIM21 (Ro/SS-A), TRIM21						+	u	d		+		
4	RO60	60 kDa SS-A/Ro ribonucleoprotein	+			+			u			+		[235]
2	RPA3	Replication protein A 14 kda subunit					+						+	[236]
3	RPF2	Ribosome production factor 2 homolog, BXDC1	+			+						+		
2	RPL10A	60S ribosomal protein L10a		+		+						+		
2	RPL11	60S ribosomal protein L11	+	+		+			u			+		
4	RPL12	60S ribosomal protein L12	+		+	+			u	d		+		[237]
2	RPL15	60S ribosomal protein L15	+	+	+	+				d		+		
3	RPL18	60S ribosomal protein L18	+		+	+				d		+		
2	RPL22	60S ribosomal protein L22	+	+	+		+			d			+	[93]
2	RPL23A	Ribosomal protein L23a			+				u				+	
2	RPL26L1	60S ribosomal protein L26-like 1, RPL26P1			+	+					Orf9b	+		
3	RPL3	60s ribosomal protein L3 (hiv-1 tar rna-binding protein b)			+				u	d		+		
2	RPL31	60S ribosomal protein L31			+				u	d			+	
2	RPL35A	60S ribosomal protein L35a				+			u	d		+		[238]
2	RPL4	60S ribosomal protein L4				+			u	d		+		
17	RPL5	60S ribosomal protein L5	+		+	+	+	+		d		+	+	[239]
11	RPL6	60S ribosomal protein L6	+		+	+	+	+	u	d		+		[181]
9	RPL7	60S ribosomal protein L7, RPL7P32	+		+	+	+	+	u	d		+		[93]
4	RPL7A	60S ribosomal protein L7A			+	+			u	d		+		[238]
2	RPL8	60S ribosomal protein L8			+	+			u	d		+		[165]
8	RPLP0	60S acidic ribosomal protein P0	+	+	+	+	+		u	d		+		[240]
2	RPLP1	60S acidic ribosomal protein P1				+			u	d		+		[241]
4	RPLP2	60S acidic ribosomal protein P2	+		+	+	+		u	d		+	+	[241]
2	RPS15A	40s ribosomal protein S15a		+	+	+			u			+		
3	RPS18	40S ribosomal protein S18	+			+			u	d	Nsp13 Orf8 Orf10	+		[165]
3	RPS19	40S ribosomal protein S19	+							d	Orf9c		+	[238]
3	RPS2	40S ribosomal protein S2	+		+	+			u	d		+		[39]
2	RPS25	40S ribosomal protein S25		+	+				u	d	Orf8	+	+	[93]
3	RPS27A	Ubiquitin-40S ribosomal protein S27a, UBA80, UBCEP1		+		+			u	d	Nsp4 S	+		[93]
6	RPS3	40S ribosomal protein S3	+	+	+	+			u	d	Orf8	+	+	[242]
3	RPS3A	40S ribosomal protein S3a	+	+	+		+		u	d	Orf8	+	+	
3	RPS4X	40S ribosomal protein S4, X isoform	+			+				d	Orf8	+		[25]
3	RPS6	40S ribosomal protein S6			+	+			u	d	Nsp6	+		[238]
3	RPS7	40S ribosomal protein S7		+	+		+		u	d			+	[93]
2	RPS8	40S ribosomal protein S8	+			+			u	d		+		
8	RPS9	40S ribosomal protein S9	+		+	+		+		d		+		[238]
5	RPSA	40S ribosomal protein SA		+		+			u	d			+	[243]
13	RRBP1	Ribosome-binding protein 1	+			+			u	d	Orf8	+	+	
11	RRP12	RRP12-like protein				+			u			+		
4	RRP9	U3 small nucleolar RNA-interacting protein 2			+	+			u	d	N	+		[244]
4	RRS1	Ribosome biogenesis regulatory protein homolog				+			u			+		
5	RSL1D1	Ribosomal L1 domain-containing protein 1				+			u	d		+		
6	RUVBL1	RuvB-like 1, tata box-binding protein-interacting protein		+	+	+					Nsp1 Nsp7 Nsp12 Orf9b	+	+	[245]
5	RUVBL2	RuvB-like 2		+						d	Nsp1 Nsp7 Nsp12 Orf9b		+	[246]
2	SARS	Serine-tRNA ligase, cytoplasmic, SARS1		+					u		Nsp15		+	
4	SEPHS1	Selenide, water dikinase	+							d			+	[247]
2	SEPT11	Septin-11		+						d			+	[25]
2	SEPT2	Septin-2, NEDD5, DIFF6	+	+					u	d			+	[248]
3	SEPT7	Septin-7		+						d			+	[249]
5	SERPINB1	Leukocyte elastase inhibitor				+			u				+	
4	SERPINB6	Serpin B6, peptidase inhibitor 6				+							+	
8	SERPINB9	Serpin B9		+					u	d			+	
2	SERPINC1	Antithrombin-III		+		+			u				+	
3	SERPINE1	Plasminogen activator inhibitor 1	+						u	d	Orf8		+	[250]
4	SERPINH1	Serpin H1, HSP47	+							d			+	[251]
6	SET	SET nuclear proto-oncogene (Inhibitor of granzyme A-activated DNase, HLA-DR-associated protein II)	+	+	+	+	+	+	u	d		+	+	[252]
2	SF3A1	Splicing factor 3 subunit 1 (spliceosome-associated protein 114) (sap 114) (sf3a120)			+				u				+	
14	SF3B1	Splicing factor 3B subunit 1	+			+			u	d		+		[253]
13	SF3B3	Splicing factor 3B subunit 3, SAP130	+	+	+	+	+	+	u			+	+	[253]
8	SFN	14-3-3 protein sigma, Stratifin				+		+	u	d			+	[254]
3	SFPQ	Splicing factor, proline- and glutamine-rich	+	+					u	d			+	[255]
3	SGTA	Small glutamine-rich tetratricopeptide repeat-containing protein alpha		+					u	d	M		+	
3	SH3BGRL3	SH3 domain-binding glutamic acid-rich-like protein 3	+							d			+	
2	SHMT1	Serine hydroxymethyltransferase, cytosolic		+						d			+	
9	SHMT2	Serine hydroxymethyltransferase, mitochondrial		+						d			+	
2	SKP1	S-phase kinase-associated protein 1	+						u	d			+	
2	SLC1A5	Neutral amino acid transporter B, Simian type D retrovirus receptor, Baboon M7 virus receptor				+			u	d	Orf3 S	+		
2	SLC2A1	HepG2 glucose transporter, GLUT1				+				d	Nsp8	+		[256]
17	SLC3A2	4F2 cell-surface antigen heavy chain, CD98	+			+			u	d	Orf3b Orf9c	+		
2	SLIRP	SRA stem-loop-interacting RNA-binding protein, mitochondrial		+					u	d			+	
4	SMS	Spermine synthase	+						u	d	Orf3		+	
9	SND1	Staphylococcal nuclease domain-containing protein 1	+	+		+			u	d			+	
15	SNRNP200	U5 small nuclear ribonucleoprotein 200 kDa helicase	+			+				d		+		[257]
3	SNRNP70	U1 small nuclear ribonucleoprotein 70 kDa		+	+		+		u	d			+	[258]
3	SNRPA	U1 small nuclear ribonucleoprotein A	+	+		+			u				+	[259]
8	SNRPA1	U2 small nuclear ribonucleoprotein A’		+	+							+	+	[260]
3	SNRPB	SnRNP-associated proteins B and B’	+		+	+			u	d		+		[261]
2	SNRPD1	Small nuclear ribonucleoprotein Sm D1	+	+		+			u			+	+	[262]
4	SNRPD2	Small nuclear ribonucleoprotein Sm D2	+	+	+	+	+			d		+	+	[263]
2	SNRPD3	Small nuclear ribonucleoprotein Sm D3	+	+			+			d		+	+	[262]
2	SNRPE	Small nuclear ribonucleoprotein E	+	+	+					d		+	+	[264]
2	SNRPG	Small nuclear ribonucleoprotein G, PBSCG				+						+		[264]
2	SOD1	Superoxide dismutase [Cu-Zn]				+			u	d			+	[265]
46	SPTAN1	Spectrin alpha chain, brain (spectrin, non-erythroid alpha chain)	+	+	+	+		+	u	d		+	+	[266]
29	SPTBN1	Spectrin beta chain, brain	+		+	+			u	d		+		[267]
3	SRM	Spermidine synthase		+						d			+	
3	SRP14	Signal recognition particle 14 kDa protein		+					u	d	Nsp13 Orf8		+	
2	SRP68	Signal recognition particle 68 kda protein			+						N Nsp2		+	
2	SRP72	Signal recognition particle 72 kDa protein				+				d	Nsp8	+		[268]
2	SRP9	Signal recognition particle 9 kda protein			+				u	d		+		
2	SRRT	Arsenite-resistance protein 2					+			d			+	
5	SRSF1	Serine/argine-rich splicing factor 1		+	+	+	+		u	d		+	+	[269]
2	SRSF11	Arginine/serine-rich splicing factor 11, SRSF11	+						u	d			+	
3	SRSF2	Arginine/serine-rich splicing factor 2, SFRS2	+	+	+				u	d			+	[65]
2	SRSF3	Serine/arginine-rich splicing factor 3, SFRS3					+						+	[270]
4	SRSF4	Splicing factor, arginine/serine-rich 4 (srp75)			+								+	
2	SRSF5	Serine/arginine-rich splicing factor 5, SRP40			+		+		u	d			+	[271]
2	SRSF6	Splicing factor, arginine/serine-rich 6			+				u	d			+	
3	SRSF7	Serine /arginine-rich splicing factor 7, SRSF7	+		+		+	+	u			+	+	[271]
2	SRSF8	Serine/arginine-rich splicing factor 8			+		+			d			+	
11	SSB	Lupus la protein (sjoegren syndrome type b antigen) (La/SSB)	+	+	+	+	+	+	u	d		+	+	[41]
9	SSBP1	Single-stranded DNA-binding protein, mitochondrial	+	+		+					N	+		
8	SSRP1	Fact complex subunit ssrp1 (facilitates chromatin transcription complex subunit ssrp1) (factp80) (chromatin- specific transcription elongation factor 80 kda)	+		+	+			u	d		+		[272]
6	ST13	Hsc70-interacting protein (hip) (suppression of tumorigenicity protein 13) (putative tumor suppressor st13) (protein fam10a1) (progesterone receptor-associate)	+	+	+	+	+		u		Nsp12 Orf3b Orf6 Orf8 Orf10		+	[273]
3	STIP1	Stress-induced-phosphoprotein 1		+					u	d	E Nsp12 Orf3a Orf8		+	[14]
2	STRBP	Spermatid perinuclear RNA-binding protein	+								Nsp15		+	
4	SUB1	Activated RNA polymerase II transcriptional coactivator p15 (PC4, RPO2TC1)	+		+			+	u	d		+	+	
2	SUGT1	Protein SGT1 homolog (Suppressor of G2 allele of SKP1 homolog)		+					u		Nsp12 Nsp15		+	
2	SUMO1	Small ubiquitin-related modifier	+							d			+	[274]
9	SUPT16H	FACT complex subunit SPT16	+		+	+				d		+		
2	SUPT5H	Transcription elongation factor SPT5				+						+		
2	SWAP70	Switch-associated protein 70		+						d	Nsp2		+	
11	TALDO1	Transaldolase		+		+			u	d			+	[275]
3	TBCA	Tubulin-specific chaperone A		+							Nsp11		+	
3	TCL1A	T-cell leukemia/lymphoma protein 1A		+					u	d			+	
7	TCP1	T-complex protein 1 subunit alpha (tcp-1-alpha) (cct-alpha)		+	+		+			d	Orf10		+	[51]
4	TEX10	Testis-expressed protein 10				+						+		
3	TFG	TRK-fused gene protein	+			+							+	
4	TGM2	Protein-glutamine gamma-glutamyltransferase 2				+			u	d			+	[276]
9	THBS1	Thrombospondin-1	+						u	d			+	[277]
29	TLN1	Talin-1	+	+	+	+			u	d			+	[278]
4	TLN2	Talin-2	+						u				+	
6	TNC	Tenascin C	+							d			+	[279]
5	TNPO1	Transportin-1, KPNB2		+									+	
3	TOP1	DNA topoisomerase 1 (Scl 70)		+	+	+		+	u			+		[280]
5	TP53I3	Quinone oxidoreductase				+			u	d			+	
3	TPD52L2	Tumor protein D54	+						u	d	Nsp4 Orf6		+	
2	TPI1	Triosephosphate isomerase			+					d	Nsp15		+	[53]
16	TPM1	Tropomyosin 1 alpha chain	+	+	+	+	+	+	u	d	Nsp9		+	[281]
17	TPM2	Tropomyosin beta chain	+		+	+		+	u	d			+	[25]
6	TPM3	Tropomyosin alpha-3 chain	+	+	+	+	+	+	u	d			+	[282]
20	TPM4	Tropomyosin alpha-4 chain	+	+	+	+	+	+	u	d			+	[283]
2	TPP1	Tripeptidyl-peptidase 1	+						u	d		+		
4	TPP2	Tripeptidyl-peptidase 2		+									+	
4	TPR	Nucleoprotein TPR	+						u	d			+	[284]
4	TPT1	Tumor protein, translationally-controlled	+						u	d			+	
3	TSN	Translin				+				d			+	
3	TTLL12	Tubulin-tyrosine ligase-like protein 12		+	+					d			+	[285]
2	TTLL3	Tubulin monoglycylase TTLL3		+					u				+	
4	TUBA1C	Tubulin alpha-1C, tubulin alpha-6	+	+	+	+	+	+	u	d		+	+	[286]
10	TUBA3C	Tubulin alpha-3C chain, TUBA2			+		+					+	+	
12	TUBA4A	Tubulin alpha-4A chain, TUBA1	+	+		+			u	d		+	+	[287]
7	TUBB	Tubulin beta chain, TUBB5	+	+	+		+		u	d		+	+	[288]
4	TUBB1	Tubulin beta-1 chain	+	+	+							+	+	[289]
2	TUBB4A	Tubulin beta-4A chain, TUBB4, TUBB5		+					u	d			+	[290]
12	TUBB4B	Tubulin beta-4B chain, TUBB2C	+	+		+		+	u	d		+	+	[289]
2	TXN	Thioredoxin	+						u	d			+	[291]
2	TXNDC17	Thioredoxin domain-containing protein 17	+	+					u	d			+	
4	TXNDC5	Thioredoxin domain-containing protein 5	+			+			u	d			+	
2	TXNL1	Thioredoxin-like protein 1 (32 kda thioredoxin-related protein)			+				u				+	
15	TXNRD1	Thioredoxin reductase 1, cytoplasmic	+	+		+			u	d			+	[291]
2	U2AF2	Splicing factor U2AF 65 kDa subunit		+						d			+	{Imai, 1993 #256}
15	UBA1	Ubiquitin-like modifier-activating enzyme 1	+	+	+	+	+	+	u	d			+	[292]
2	UBA2	Ubiquitin-like 1-activating enzyme e1b (sumo-1-activating enzyme subunit 2) (anthracycline-associated resistance arx)			+					d	Nsp7		+	
2	UBA6	Ubiquitin-like modifier-activating enzyme 6		+							Nsp7		+	
2	UBE2K	Ubiquitin-conjugating enzyme E2 K		+									+	
2	UBLE1A	Ubiquitin-like 1-activating enzyme e1a (SUMO-1-activating enzyme subunit 1), SAE1	+		+				u	d			+	[274]
2	UBTF	Nucleolar transcription factor 1, autoantigen NOR-90				+				d		+		[293]
2	UCHL1	Ubiquitin carboxyl-terminal hydrolase isozyme L1	+			+			u	d	Nsp7 Orf3		+	[294]
5	UGDH	UDP-glucose 6-dehydrogenase				+			u	d			+	
6	UGGT1	UDP-glucose:glycoprotein glucosyltransferase 1, UGCGL1	+							d	Orf3a Orf7a		+	
18	UPF1	Regulator of nonsense transcripts 1	+			+				d	N	+		
3	USP5	Ubiquitin carboxyl-terminal hydrolase 5 (ubiquitin thioesterase 5) (ubiquitin-specific-processing protease 5) (deubiquitinating enzyme 5) (isopeptidase T)	+	+	+				u	d			+	
2	USP7	Ubiquitin carboxyl-terminal hydrolase (Herpes virus associated)				+			u		E M Nsp4 Orf7a Orf7b	+		
2	USP9X	Ubiquitin specific protease 9, X chromosome	+						u	d		+		
3	VARS1	Valine-tRNA ligase		+									+	
4	VASN	Vasorin	+						u	d			+	
4	VAT1	Synaptic vesicle membrane protein VAT-1 homolog	+						u	d	Orf3b Orf6		+	
27	VCL	Vinculin	+			+			u	d	Nsp14		+	[295]
18	VCP	Transitional endoplasmic reticulum ATPase (Valosin-containing protein) (ER)	+	+	+	+	+	+	u	d			+	[296]
17	VIM	Vimentin	+	+	+	+	+	+	u	d		+	+	[297]
2	VPS35	Vacuolar protein sorting 35					+		u	d			+	[298]
6	WARS	Tryptophanyl-tRNA synthetase, cytoplasmic	+	+					u	d			+	[299]
5	WDR18	WD repeat-containing protein 18				+				d	Nsp15	+		
2	XPNPEP1	Xaa-Pro aminopeptidase 1		+	+					d			+	
4	XPO1	Exportin-1		+							Nsp4 Orf7a		+	
10	XPO2	Exportin-2, CAS, CSE1L		+						d			+	
5	XPOT	Exportin-T (trna exportin) (exportin(trna))		+	+				u		Orf7a		+	
32	XRCC5	ATP-dependent DNA helicase 2 subunit 2, Ku80	+	+	+	+	+	+		d		+	+	[300]
30	XRCC6	ATP-dependent DNA helicase 2 subunit 1, Ku70	+	+	+	+	+	+	u	d		+	+	[300]
6	YARS	Tyrosine-tRNA ligase, cytoplasmic, YARS1		+					u	d			+	[301]
3	YBX1	Y-box-binding protein 1			+	+			u	d		+		[302]
6	YBX3	Y-box-binding protein 3	+		+	+		+	u	d		+		[303]
12	YWHAB	14-3-3 protein beta/alpha	+	+	+	+	+	+	u	d			+	
15	YWHAE	14-3-3 protein epsilon	+	+	+	+	+	+	u	d			+	[254]
6	YWHAG	14-3-3 protein gamma	+	+	+	+	+	+	u	d			+	[254]
5	YWHAH	14-3-3 protein eta	+	+	+	+	+			d			+	[304]
7	YWHAQ	14-3-3 protein theta	+	+	+	+	+	+	u	d			+	[243]
7	YWHAZ	14-3-3 protein zeta/delta	+	+	+	+	+	+	u	d			+	[305]
2	ZPR1	Zinc finger protein ZPR1		+					u	d			+	[306]

**Columns from left to right:** P (the largest number of peptides identified for a protein by mass spectrometry for all cell lines), gene symbol, protein name, cell lines (HFL1 fetal lung fibroblast, HS-Sultan B lymphoblast, Wil2-NS B-lymphoblast, A549 lung epithelial cell, Jurkat T-lymphoblast, HEp-2 fibroblast), SARS-Cov-2 infection (up-regulated, down-regulated, interactome of specific viral protein), dermatan sulfate (DS) affinity (high affinity: eluted from DS-affinity resins with 1.0 M NaCl; low affinity: eluted with 0.4–0.6 M NaCl), Ref. (representive paper reporting autoantibodies specific for the autoAg protein). Highlighted in red: common (shared) autoAgs found in all 6 cell lines.

**Table 2.** Diseases associated with potential SARS-CoV-2 spike protein-interacting autoAgs*

CALU	Warfarin (anti-coagulants for blot clotting) resistance disease
ESYT1	Stormorken syndrome (mild bleeding tendency due to platelet dysfunction, thrombocytopenia, anemia, asplenia, etc.)
MOV10	Viral infection, autism spectrum disorder
MARCKS	Spinocerebellar ataxia 14, barbiturate dependence
HSPBP1	Autosomal recessive spinocerebellar ataxia 16, Sjögren-Larsson syndrome
PRS27A	Machado-Joseph disease (spinocerebellar ataxia type III), spherocytosis type 5
EZR	Autosomal recessive non-syndromic intellectual disability, neurofibromatosis type II
HSPA5	Mucormycosis (fungal infection), Wolfram syndrome 1 (diabetes mellitus)
ARHGAP1	Noma, Lowe oculocerebrorenal syndrome (affects eyes, central nervous system and kidneys)
MSN	X-linked moesin-associated immunodeficiency, verrucous carcinoma
CSPG4	Acral lentiginous melanoma, melanoma
SLC1A5	Hartnup disorder, placental choriocarcinoma
PRKCSH	Polycystic liver disease
CAVIN1	Lipodystrophy, congenital generalized lipodystrophy
BASP1	Distal X-linked spinal muscular atrophy, Wilms tumor 1

* Disease associations were obtained from the GeneCards database.
